# Injectable ε‑Polylysine/Hyaluronic Acid Hydrogels with Resistance-Preventing Antibacterial Activity for Treating Wound Infections

**DOI:** 10.1021/acsabm.5c01252

**Published:** 2025-10-30

**Authors:** Artemijs Sceglovs, Claudia Siverino, Ingus Skadins, Marika Sceglova, Valdis Pirsko, Thomas Fintan Moriarty, Juta Kroica, Kristine Salma-Ancane

**Affiliations:** † Institute of Biomaterials and Bioengineering, Faculty of Natural Sciences and Technology, 87254 Riga Technical University, Paula Valdena St. 3, Riga lv-1048, Latvia; ‡ Baltic Biomaterials Centre of Excellence, Headquarters at Riga Technical University, Paula Valdena St. 3, K-1, Riga lv-1048, Latvia; § 58065 AO Research Institute Davos, Clavadelerstrasse 8, Davos 7270, Switzerland; ∥ Department of Biology and Microbiology, 87255 Riga Stradins University, Dzirciema St. 16 Riga lv-1007, Latvia; ⊥ Institute of Microbiology and Virology, Riga Stradins University, Ratsupites St. 5, Riga lv-1067, Latvia

**Keywords:** antibacterial hydrogels, ε-polylysine, hyaluronic acid, multidrug-resistant bacteria, resistance prevention, antibiofilm

## Abstract

The growing threat of antimicrobial resistance has created an urgent demand for nonantibiotic biomaterials capable of preventing infections without promoting bacterial resistance. In this study, we developed injectable, covalently cross-linked hydrogels composed of ε-poly-l-lysine (ε-PL) and hyaluronic acid (HA) for localized wound infection treatment. These hydrogels combine the inherent antibacterial properties of ε-PL with the biocompatibility of HA, forming a shear-thinning, self-recovering system suitable for syringe-based administration. We first evaluated the antibacterial activity of pure ε-PL, determining minimum inhibitory and bactericidal concentrations (MIC/MBC) and evaluating resistance development against ATCC and clinically isolated multidrug-resistant strains (MRSA, ESBL-*E. coli*, *P. aeruginosa*). Notably, no resistance emerged in any strain after the serial passages. Hydrogels formed at varying ε-PL/HA ratios demonstrated strong immediate and long-term bactericidal activity while maintaining high cytocompatibility with murine and human fibroblasts. The hydrogels significantly reduced biofilm formation of *S. aureus* and MRSA within 24 h, achieving reductions comparable to or greater than vancomycin-gentamicin controls. Rheological analysis confirmed injectability, stability, and tunable stiffness. This study presents the first demonstration that ε-PL-based hydrogels can prevent resistance development in multidrug-resistant pathogens, offering a safe and antibiotic-free approach for infection control. The combination of antibacterial efficacy, resistance prevention, and biocompatibility makes these hydrogels promising candidates for wound infection management.

## Graphical abstract

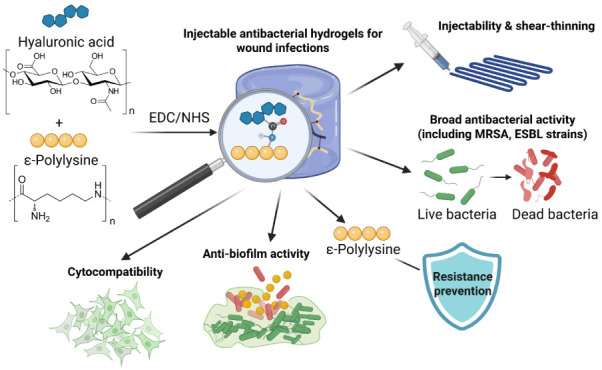

## 1 Introduction

Bacterial infections account for 13.6% of global mortality, and the rise of antimicrobial resistance (AMR) represents a major global health crisis due to the growing prevalence of multidrug-resistant pathogens.^1–3^ In 2019, six priority AMR pathogens (including extended-spectrum β-lactamase-producing *Escherichia coli* (ESBL *E. coli*), methicillin-resistant *Staphylococcus aureus* (MRSA), etc.) were linked to ∼930,000 direct deaths and associated with 3.57 million deaths.^4,5^ By 2050, AMR is projected to be associated with more than 30 million deaths worldwide.^6^ The WHO Global Action Plan has emphasized the urgent need to develop alternative, nonantibiotic therapies.^7,8^ Healthcare-associated infections (e.g., surgical sites, infected wounds, implants) often require systemic antibiotic therapy, which is ineffective due to biofilm formation and poor antibiotic penetration.^9,10^ Antibiotic-loaded commercial biomaterials for wound infection treatment are available in clinics and can enhance the local drug concentration. However, they may still promote resistance and adverse tissue responses.^10^ Commercially available nonantibiotic wound dressings, including silver- or iodine-based products, are also used for tissue infection treatment, but their long-term effectiveness is limited and may cause cytotoxicity. This highlights the urgent need to develop safe, sustained nonantibiotic therapies for localized treatment. Injectable hydrogels have been extensively explored for localized infection treatment, as they can be applied directly into wounds or deep tissues and provide sustained, targeted release of antibacterial agents.^11^ A range of nonantibiotic antibacterial agents have been incorporated into such hydrogels, including metal and metal oxide nanoparticles,^12,13^ enzymes,^14^ bacteriophages,^15^ and especially antimicrobial peptides (AMPs). AMPs, from both natural and synthetic sources, are particularly attractive due to their broad-spectrum activity against multidrug-resistant bacteria.^16^ They kill bacteria primarily by disturbing membranes through electrostatic interactions, along with additional mechanisms, which makes resistance development less likely than with conventional antibiotics.^15,16^ However, their precise modes of action are not yet fully understood.^17^

Biomaterial-based AMP delivery systems have shown promise for infection prevention and treatment, including applications in wound healing,^17–21^ implant coatings,^22^ and advanced carriers such as microneedles,^23^ dressings,^22,24^ nanoparticles,^24,25^ and hydrogels.^21,22^ However, these systems face several functional challenges, including limited loading capacity, burst release, and reduced long-term efficacy and safety.^11,25^ A promising alternative is to integrate AMPs directly into a 3D hydrogel network, creating inherent antibacterial properties through surface chemistry and molecular design.^22,23^ This strategy provides stable, localized, and sustained activity, including inhibition of bacterial growth, membrane disruption, and biofilm prevention,^11,16,24^ while allowing cross-linking and AMP content to be tuned to balance biodegradation, efficacy, and biological safety.

In recent years, naturally derived AMP (nAMP)-based hydrogels (e.g., LL-37, β-defensins, hLF1–11, magainin) have emerged as promising nonantibiotic therapeutics for wound healing and tissue infection prevention.^26,27^ Their evolutionary origin contributes to lower cytotoxicity, higher biocompatibility, reduced resistance potential, and multifunctional activity compared to synthetic alternatives.^28^ While most nAMPs are short-chain, long-chain antimicrobial polypeptides such as ε-polylysine (ε-PL) are of special interest. ε-PL is a naturally occurring cationic poly­(amino acid) produced by *Streptomyces albulus*, is FDA-recognized as GRAS and has been used safely for decades as a food preservative. More recently, it has attracted attention as a broad-spectrum antibacterial biopolymer for tissue engineering applications, including wound dressings, scaffolds, coatings and hydrogels.^29–32^ Its strong positive charge and simple lysine-based structure provide biocompatibility, low toxicity, low immunogenicity, and biodegradability.^29–32^ At physiological pH, the ε-amino side groups of lysine residues are protonated to −NH_3_^+^, giving ε-PL its strong polycationic character. These positively charged groups interact electrostatically with bacterial surface components, such as lipopolysaccharides in Gram-negative and lipo-/teichoic acids in Gram-positive bacteria, resulting in bacterial membrane disruption, metabolic interference, and reactive oxygen species (ROS) induction.^33,34^ Despite promising preclinical data, no ε-PL-based medical products exist, and only a few hydrogels have been tested in preclinical studies.^14,35,36^ Examples include polyglutamic acid/ε-PL composites,^37^ silk fibroin/ε-PL hydrogels,^38^ and polyacrylamide/gelatin/ε-PL hydrogels,^39^ among others.^40,41^ However, no previous studies have systematically evaluated the antibacterial activity of ε-PL-based hydrogels against clinically relevant multidrug-resistant pathogens nor investigated their potential for resistance development or biofilm inhibition, key translational benchmarks addressed in the present study. To date, no clinically available ε-PL-based dressings exist and HA-based hydrogels alone are inefficient for infected wounds. This study of ε-PL/HA hydrogels directly addresses this gap by combining the inherent antibacterial activity of ε-PL with the wound healing activity of HA, thereby advancing the translational potential of nonantibiotic therapeutics for treating multidrug-resistant infections. In our previous studies, covalently cross-linked ε-PL/HA hydrogels were developed using EDC/NHS-mediated carbodiimide chemistry.^42,43^ These hydrogels exhibited bactericidal activity against ATCC reference strains of *E. coli* and *S. aureus*, assessed by the agar diffusion method.

The aim of this study was to evaluate the clinically relevant antibacterial potential of ε-PL and antibacterial activity, cytocompatibility, rheology/injectability, and antibiofilm performance of covalently cross-linked ε-PL/HA hydrogels for minimally invasive, syringe-based delivery to infection sites. To systematically investigate the impact of ε-PL content on *in vitro* cytocompatibility and antibacterial efficacy, three ε-PL/HA hydrogel series 50:50, 60:40, and 70:30 wt % were selected. These series were chosen based on our previous studies, where they demonstrated a validated balance between inherent antibacterial activity and mechanical robustness, making them suitable for local infection treatment applications.^42,43^

The antibacterial performance of pure ε-PL was first evaluated through minimum inhibitory concentration (MIC) and minimum bactericidal concentration (MBC) testing and the evaluation of bacterial resistance development against ATCC reference *E. coli*, *Staphylococcus aureus* (*S. aureus*), and *S. epidermidis*, clinically isolated *P. aeruginosa* and the multidrug-resistant pathogens MRSA and ESBL *E. coli*. Subsequently, the bactericidal activity of the hydrogels was evaluated against the same ATCC reference strains and clinically isolated multidrug-resistant bacterial strains. In addition, the antibiofilm activity of the hydrogels was evaluated against *S. aureus* and *MRSA*. The cytocompatibility was evaluated by using human dermal fibroblasts (HDFs) and Balb/c 3T3 fibroblasts. This study provides the first evidence that ε-PL/HA hydrogels exhibit strong bactericidal performance and antibiofilm activity, while pure ε-PL does not induce bacterial resistance, highlighting their potential in bacterial wound infection treatment.

## 2 Materials and Methods

### 2.1 Materials

ε-PL (ε-PL·HCl, 99% purity, molecular weight 3850 g/mol, humidity content 6.5%) was purchased from Zhengzhou Bainafo Bioengineering Co., Ltd. (Henan, China). Sodium hyaluronate (Na-HA, 95% purity, humidity content 13.5%, molecular weight 1.55 MDa) was purchased from Contipro Biotech s.r.o. (Dolní Dobrouč, Czech Republic). 1-Ethyl-3-(3-(dimethylamino)­propyl)-carbodiimide hydrochloride (EDC, 98% purity, CAS No.: 25952-53-8, molecular weight: 191.75 g/mol) was purchased from Novabiochem (Burlington, USA). *N*-Hydroxysuccinimide (NHS, 98% purity, CAS No.: 6066-82-6, molecular weight: 115.09 g/mol) was purchased from Sigma-Aldrich.

*E. coli* ATCC 25922, Methicillin-sensitive *S. aureus* ATCC 25923, and *S. epidermidis* ATCC 35984 were acquired from the *American Type Culture Collection* (ATCC, USA). *P. aeruginosa* was a clinical isolate cultured from a patient with a biofilm-related infection (St. Gallen Kantonsspital, Switzerland). Methicillin-resistant *S. aureus* (*MRSA*) was isolated from a patient pus sample (Riga Stradins Hospital, Latvia). *ESBL* *E. coli* was clinically isolated from patients (Riga Stradins Hospital, Latvia). Tryptone soy broth (TSB, CM0129) was purchased from Oxoid Limited (Hampshire, United Kingdom). Lysogeny broth (LB, Cat. Nr. 1102850500) was purchased from *Merck KGaA* (Darmstadt, Germany). Tryptone Soya Agar (TSA, casein soybean digest agar, Code: CM0131) was purchased from *Oxoid Limited* (Hampshire, United Kingdom).

For the cell culture experiments, human dermal fibroblasts (HDFs) from Thermo Fisher Scientific Inc. (Waltham, USA) were used. The Balb/c 3T3 mouse fibroblast cell line was obtained from the American Type Culture Collection (CCL-163, ATCC, USA). Dimethyl sulfoxide was purchased from *Labochema* (Latvia). Phosphate-buffered saline (PBS, liquid, pH 7.2) was obtained from *Sigma-Aldrich*. Trypsin (Trypsin-EDTA (0.25%), phenol red); Penicillin-Streptomycin (PenStrep, 10,000 U/mL); Dulbecco’s Modified Eagle Medium/Nutrient Mixture F-12 (DMEM/F12; Cat# 11320033); Fetal Bovine Serum (FBS); Calcein (AM, cell-permanent dye); and Dulbecco’s Modified Eagle Medium (DMEM) were purchased from *Thermo Fisher Scientific Inc.* (Waltham, USA); The CellTiter Blue cell viability assay was purchased from *Promega Corporation* (Madison, USA); LIVE/DEAD staining with Hoechst 33342 was purchased from *Sigma-Aldrich* (5 μg/mL, Cat# 14533), and propidium iodide was purchased from *Invitrogen* (1 μg/mL, Cat# P1304MP)

### 2.2 Fabrication of Chemically Cross-Linked ε-PL/HA Hydrogels

The *in situ* forming covalently cross-linked ε-PL/HA hydrogel series with ε-PL to HA mass ratios of 50:50, 60:40, and 70:30 wt % were prepared via EDC/NHS-mediated carboxyl-to-amine cross-linking, step-by-step, following the synthesis methodology as previously described^43^ (Figure 1). EDC acts as an activator by reacting with the carboxyl groups (−COOH) of HA and forms an unstable O-acylisourea intermediate, which is stabilized by NHS through the formation of NHS esters. These activated esters are attacked by the primary ε-amino (−NH_2_) groups of ε-PL, leading to the formation of stable covalent amide bonds and a chemically cross-linked ε-PL/HA hydrogel network.^42,43^ The EDC to NHS molar ratio was 1:1 for all HA to ε-PL mass ratios of ε-PL/HA hydrogel series to introduce the uncross-linked primary ε-amino (−NH_2_) groups of ε-PL. Hydrogels were synthesized by mixing and homogenization components via an interconnected syringe technique (Fisher Scientific, BD PlastiPak Syringe with Luer Lock, 5 mL) at room temperature (23 °C) following the order described as follows: 1) preparation of the starting components in syringes: syringe No. 1 (S1) was prepared by rapid mixing of 0.21 g of Na-HA powder with 2 mL of deionized water (DW); the S2 and S3 contained 0.19 g of EDC and 0.11 g of NHS, respectively; the S4 was prepared by rapid mixing of the appropriate amount of ε-PL – 0.19, 0.29, and 0.46 g with 2 mL DW, corresponding to the desired ε-PL to HA composition of 50:50, 60:40 and 70:30 wt %, respectively. All prepared syringes (S1–S4) were stored in the refrigerator at 4 °C for 24 h; 2) synthesis of chemically cross-linked the ε-PL/HA hydrogels: after 24 h, the S1 containing Na-HA aqueous solution was mixed with the S2 containing EDC powder by using the same interconnected syringes. Then, the preactivated aqueous solution was mixed with the S3 containing NHS. Finally, the S4 containing ε-PL solution was rapidly mixed with the preactivated HA/EDC/NHS syringe for 1 min; 3) fabrication of as-prepared hydrogel 3D samples: as-prepared hydrogels were cast into cylindrical molds (Ø 10 mm, H 5 mm) and left for complete cross-linking for 24 h at room temperature (23 °C). For *in vitro* studies, as-prepared hydrogel samples were steam-sterilized in an autoclave at 121 °C for 20 min under 215 kPa pressure. The designation and composition of the ε-PL/HA hydrogel series are summarized in Table 1.

**Figure 1:**
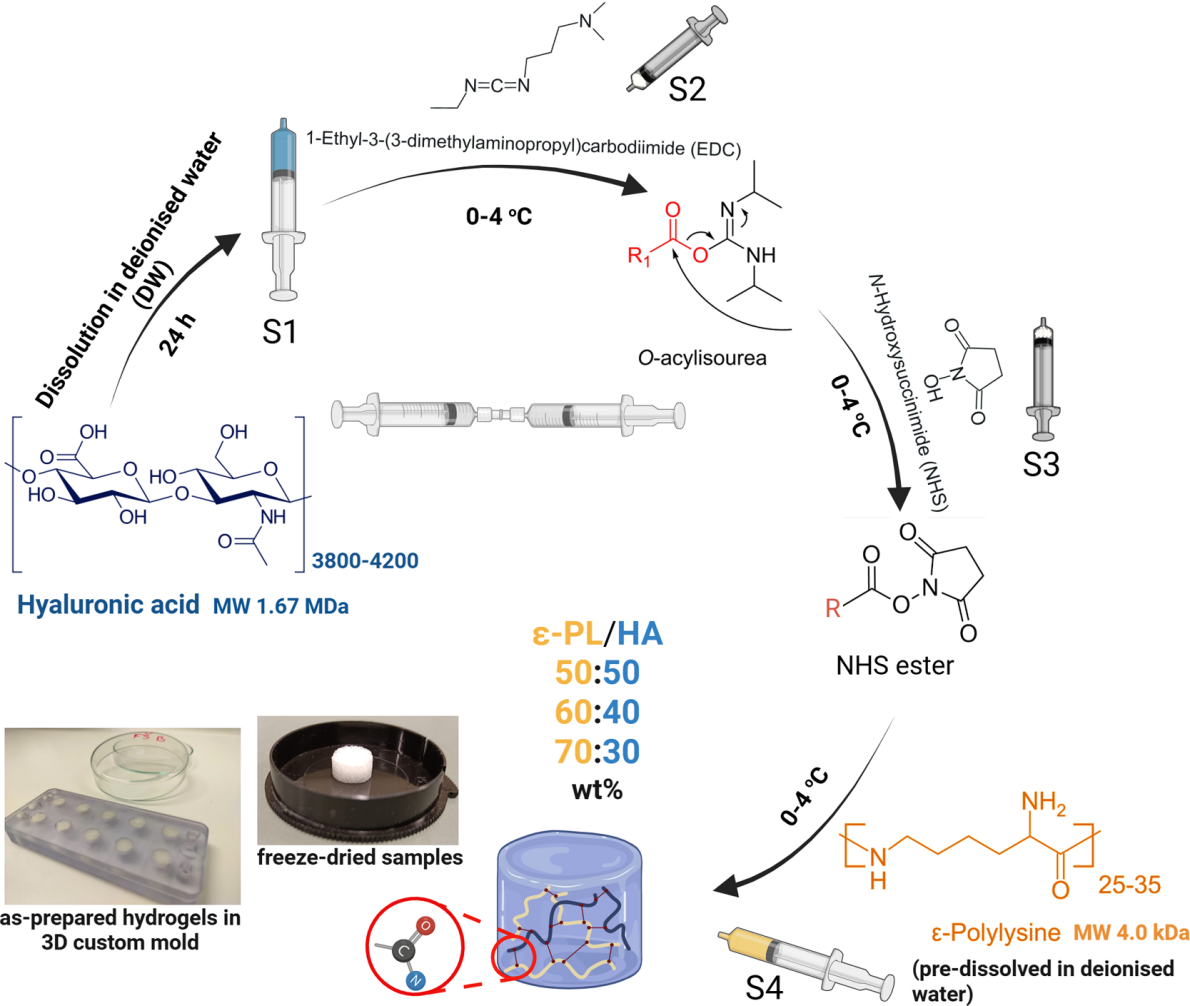
Schematic representation of the synthesis route of chemically cross-linked ε-PL/HA hydrogels.

**Table 1:** Designation and Composition of the Synthesized Chemically Crosslinked ε-PL/HA Hydrogels

Designation	ε-PL to HA mass ratio, wt %	ε-PL (−NH_2_), mmol	HA (−COOH), mmol	EDC:NHS, molar ratio	Liquid volume, mL
**ε-PL/HA 50:50** wt %	50:50	0.0479	0.000119	1:1	4
**ε-PL/HA 60:40** wt %	60:40	0.0718	0.000119	1:1	4
**ε-PL/HA 70:30** wt %	70:30	0.0838	0.000119	1:1	4

### 2.3 Rheological Studies

Temperature-dependent viscosity, shear rate-dependent viscosity, and recovery cycle tests, as well as time and amplitude sweep studies, were chosen for rheological studies to evaluate the injectability, shear-thinning, and self-healing features of the ε-PL/HA hydrogels. These rheological studies were performed to investigate whether the hydrogels could be applied by minimally invasive delivery to bacterial infection sites via syringe extrusion. Time sweep measurements were conducted to determine the gelation time of the as-prepared ε-PL/HA hydrogels, which is a critical parameter for injectable biomaterials. Gelation time was evaluated by monitoring changes in the storage modulus and axial force over time. The experiment was carried out for 215 min under LVR conditions (0.2% strain and 1 Hz) and physiological temperature (37 °C). Unlike subsequent rheological studies, the hydrogel samples in this test were loaded into the rheometer immediately after syringe mixing, using a 25 mm geometry plate and a 1300 μm gap, without allowing any relaxation time. Amplitude sweep studies were conducted to reveal the cross-linking degree of the prepared hydrogels by extracting the storage modulus from the linear viscoelastic region (LVR) of amplitude sweep curves of the ε-PL/HA hydrogel series. Extracted storage modulus values were added to the equation. Finally, cross-linking densities (*q*, mol·m^–3^) were obtained: *q = Mw/Mc*, where *Mw* is the molecular weight of the cross-linked monomer calculated as follows: *Mw = Mw­(HA) + Mw­(ε-PL)*, where *Mw­(HA)* is the molecular weight of a HA monomer, and *Mw­(ε-PL)* is the molecular weight of an ε-PL monomer. In turn, *Mc*, e.g., molecular weight between cross-links of the prepared hydrogels was calculated as *Mc = RTd/G′*, where *R* is the universal gas constant (8.314 m^3^ × Pa × K^–1^ × mol^–1^), *T* is the absolute temperature (298 K), and *d* is the density of the polymer (found experimentally as *d = m/V = m/πr* ^2^ *h*), *G′* is a storage modulus at 0.2% strain and 1 Hz frequency according to the defined LVR.^44^ The Thermo HR-20 Hybrid rheometer from TA Instruments (USA) was used to perform rheological characterization. A 25 mm parallel plate with a gap of 2.0–3.0 mm was used in all cases. Silicone oil was gently applied around the sample to avoid evaporation, and a humidity control trap was used. Before each measurement, each sample was left to a relaxation time of 180 s. The sample loading procedure was followed step-by-step according to the methodology described in a previous report.^43,44^ Temperature-dependent viscosity tests were done in flow mode at a temperature range of 4 to 37 °C. Viscosity values were measured at each 1 °C step change. During shear rate-dependent viscosity tests, shear rate values were changed logarithmically from 0.5 to 500 s^–1^. Finally, in recovery tests, the viscosity values were measured over five cycles. The cycle parameters were as follows: first, third, and fifth cycles—constant shear rate of 0.1 s^–1^ for 60 s; second and fourth cycles—rapidly increased constant shear rate of 200 s^–1^ for 10 s. To obtain amplitude sweep curves of the prepared ε-PL/HA hydrogels, studies were performed in oscillation mode at a constant frequency (1 Hz) and temperature (25 °C). Amplitude strain modulus values were changed logarithmically during the test from 0.01% to 1000%. All measurements were repeated three times to ensure reproducibility.

### 2.4 *In Vitro* Antibacterial Activity Assay

A series of antibacterial tests was designed to investigate the antibacterial performance of the ε-PL/HA hydrogels and the pure antimicrobial polypeptide ε-PL. First, the minimum inhibitory concentration (MIC), minimum bactericidal concentration (MBC), and bacterial resistance development against ε-PL were evaluated. Additionally, antibiofilm studies, as well as both direct bactericidal activity and indirect assessments of antibacterial activity, were performed for the as-prepared (hydrated form) ε-PL/HA hydrogels. The results of these investigations are detailed in the following sections.

#### 2.4.1 Minimum Inhibitory/Bactericidal Concentration Studies of ε-PL

The MIC and MBC studies were performed for ε-PL aqueous solutions against target *E. coli*, *S. aureus*, *S. epidermidis*, *P. aeruginosa*, *ESBL* *E. coli*, and *MRSA* listed in Section 2.1 The bacteria were cultured overnight in 20 mL of TSB at 37 °C with agitation at 60 rpm. Overnight cultures were diluted in TSB to an optical density at 600 nm (OD_600_) of 0.1, corresponding to a bacterial concentration of approximately 1–2 × 10^8^ colony-forming units (CFUs). Bacteria (10 μL from the OD = 0.1 corresponding to 1 × 10^6^ CFU/mL) were then incubated for 24 h (60 rpm and 37 °C) at a range of concentrations of ε-PL aqueous solution (4, 8, 18, 37, 75, 150, 200, 350, 400, 500, and 600 μg/mL). After incubation with hydrogels, bacterial suspensions were collected from each well and serially diluted 10-fold (10^0^ to 10^–6^) in sterile PBS. Aliquots of 10 μL from each dilution were spotted onto tryptic soy agar (TSA) plates and incubated at 37 °C for 24 h under static conditions. Colony-forming units (CFUs) were then enumerated from the dilution, yielding countable colonies.

#### 2.4.2 Evaluation of Bacterial Resistance Development against ε-PL

*E. coli*, including ESBL *E. coli*, as well as *S. aureus*, including MRSA, were used in this study. In the beginning, the bacteria were cultured overnight, then diluted to an OD of 0.1 and incubated with the experimental range of ε-PL aqueous solution exactly as described above for MIC studies. The experimental concentration range of the ε-PL aqueous solutions was chosen based on MIC values for each strain ±1–2 fold concentrations and turned out to be the same (control, 8, 18, 37, 75, and 150 μg/mL). To assess resistance development, each strain was passaged twice weekly in the presence of subinhibitory concentrations of ε-PL. The passaging process included inoculating surviving bacterial colonies into 20 mL of broth media for 24 h at 37 °C and 60 rpm. Afterward, bacteria were transferred, streaked on a TSA plate, and incubated for 24 h at 37 °C. MICs were determined using the broth microdilution method according to CLSI guidelines.^45^ MICs were recorded as the lowest concentration of ε-PL that prevented visible growth after 24 h (37 °C) of incubation, and MIC determination was performed after passages 1, 2, 4, 6, and 10 to track the development of resistance. Three parallel replicates from each concentration were studied to ensure result validation.

#### 2.4.3 Antibiofilm Activity of ε-PL/HA Hydrogels

In order to investigate the antibiofilm activity of the prepared ε-PL/HA hydrogels, two studies were performed: antibiofilm activity via biofilm biomass quantification using the crystal violet assay and antibiofilm activity with the Live/Dead assay and quantitative viability.

##### 2.4.3.1 Biofilm Biomass Quantification via Crystal Violet Assay

The antibiofilm activity of the ε-PL/HA hydrogels was evaluated using the crystal violet assay^46–48^ against two of the most common healthcare-associated biofilm-forming bacteria, *S. aureus* and *MRSA*.^49–51^ Overnight bacterial cultures were diluted 1:50 in TSB (corresponding to a bacterial concentration of 6–7 × 10^7^ CFU/mL), and 24-well plates were inoculated with 1 mL of the suspension and incubated for 24 h at 37 °C under static conditions to allow biofilm formation. Following incubation, the medium was removed and wells were assigned to three groups: (i) untreated controls (1 mL of fresh b suspension, *n* = 4); (ii) antibiotic-treated controls (1 mL of vancomycin/gentamicin mixture, 3 and 2 mg/mL, respectively; *n* = 4); (iii) hydrogel-treated wells (one hydrated hydrogel disc, *h* = 5 mm, ø = 10 mm, ∼0.4 g, immersed in 1 mL of 0.9% NaCl, *n* = 4). All plates were sealed with parafilm and incubated for an additional 24 h at 37 °C under static conditions. After treatment, hydrogels were removed, and wells were washed three times with 1 mL of 0.9% NaCl. Biofilms were stained with 1 mL of 0.1% crystal violet for 20 min, rinsed once with 0.9% NaCl, and decolorized with 1 mL of 96% ethanol. Three 100 μL aliquots per well were transferred to a 96-well plate, and absorbance was measured at 570 nm using a Tecan microplate reader (*Tecan Trading AG*, Switzerland).

##### 2.4.3.2 Antibiofilm Activity of ε-PL/HA Hydrogels via Live/Dead and Viability Assay

For proof-of-concept antibiofilm studies, Live/Dead assay experiments were performed against *S. aureus* due to its clinical relevance and biofilm-forming ability, as described in Section 2.4.3.1. Overnight bacterial cultures were diluted 1:50 in TSB, and two parallel 24-well plates were inoculated with 1 mL of the suspension and one hydrogel sample per well (dimensions as described in Section 2.4.3.1, with three replicates per composition). Plates were incubated for 24 and 72 h at 37 °C under static conditions to allow biofilm formation on the hydrogel surface. Titanium (Ti) discs of identical size were used as positive controls in each plate. For the 72 h experiment, hydrogel samples and Ti discs were transferred daily into fresh wells containing newly added TSB to support continuous biofilm growth and to remove planktonic bacteria.

After 24 h, hydrogels and Ti discs were transferred into empty wells, washed twice with 1 mL of 0.9% NaCl, and stained with 1 mL of Live/Dead dye (BacLight Bacterial Viability Kit, Component A – 1.67 mM SYTO 9 and 1.67 mM PI; Component B – 1.67 mM SYTO 9 and 18.3 mM PI; Invitrogen, Thermo Fisher, USA). Samples were incubated for 15 min in the dark to avoid photobleaching. Biofilms were then analyzed by confocal microscopy (LSM900, Zeiss AG, Feldbach, Switzerland) using two channels: Ex/Em 480/635 nm (green) and Ex/Em 535/617 nm (red). After imaging, hydrogel samples and Ti discs were placed in 2 mL Eppendorf tubes containing 1 mL of 0.9% NaCl, vortexed for 1 min at 3000 rpm, sonicated for 10 min (Bandelin electronic GmbH & Co. KG, Germany), and vortexed again for 1 min. Aliquots were transferred into 96-well plates, serially diluted 6-fold, and plated on TSA for viable bacteria enumeration, as described in Section 2.4.1. The same procedure was followed for the 72 h samples.

#### 2.4.4 Direct Test of Antibacterial Activity of ε-PL/HA Hydrogels

Bactericidal activity of the ε-PL/HA hydrogels was performed according to CLSI and EUCAST standards,^45,52^ with minor modifications in order to adjust the protocol procedure for hydrogel testing up to 24 h. Bacterial suspensions of the target bacterial strains of *E. coli*, *S. aureus*, *S. epidermidis*, clinically isolated *P. aeruginosa*, and clinically isolated multidrug-resistant *ESBL* *E. coli* and *MRSA* were prepared in a glass tube with 0.9% NaCl until they reached McFarland = 1.0, corresponding to approximately 3 × 10^8^ CFU/mL. Experimental 6-well plates were prepared for each bacterial strain, including: (i) three replicates of sterilized hydrogel samples (one scaffold (*h* 5 mm, ø 10 mm, ∼0.4 g) per well) from each series, immersed in 2 mL of bacterial suspension; (ii) a positive control contained 2 mL of pure 0.9% NaCl; and (iii) a negative control contained −2 mL of bacterial suspension in 0.9% NaCl. The prepared six-well plates were tightly tied with parafilm and incubated for 24 h at 60 rpm and 37 °C. Afterward, six-stage dilutions were prepared, and the surviving bacterial colonies were counted according to the previous description in Section 2.4.1.

#### 2.4.5 Long-Term Antibacterial Activity of ε-PL/HA Hydrogels

Long-term antibacterial studies included both direct contact tests on prepared ε-PL/HA hydrogels and indirect/supernatant studies on collected supernatants after an incubation period of 168 h implemented within the stability testing of ε-PL/HA hydrogels (Section 2.4.5.1). The hydrogels ε-PL/HA 50:50 wt %, ε-PL/HA 60:40 wt %, and ε-PL/HA 70:30 wt % were incubated in 50 mL DW (0.8% v/v) at 60 rpm and 37 °C for 168 h (with media refreshing at 1 h and 24 h).

For long-term indirect/supernatant studies: after 168 h, the supernatants were collected. Briefly, 1 mL of supernatant and 1 mL of prepared bacterial suspension (*E. coli* and *S. aureus*) in 0.9% NaCl with McFarland 1.0 (approximately 3 × 10^8^ CFU/mL) were mixed, and antibacterial activity was evaluated after 24 h at 37 °C of incubation according to the methodology described in Section 2.4.4.

For long-term direct studies: ε-PL/HA hydrogel samples (50:50, 60:40, 70:30 wt %) were extracted after 168 h incubation in DW and used for the direct test with *E. coli* (Gr−) and *S. aureus* (Gr+). Extracted hydrogel samples (one scaffold ∼0.6 g per well, triplicates from each series were used) were immersed in 2 mL of bacterial suspension of McFarland 1.0 (3 × 10^8^ CFU/mL), and the procedure following incubation for 24 h at 37 °C was followed according to the steps described in Section 2.4.4.

##### 2.4.5.1 Swelling Behavior and Structural Stability Studies of ε-PL/HA Hydrogels

In order to prepare ε-PL/HA hydrogel samples for long-term antibacterial activity studies as well as to collect supernatants for indirect testing, stability studies were conducted to reveal what happens to hydrogels in physicochemical aspects and how it affects their further antibacterial performance. Three replicates in hydrated form without freeze-drying from the hydrogels ε-PL/HA 50:50 wt %, ε-PL/HA 60:40 wt %, and ε-PL/HA 70:30 wt % series were weighed first to obtain the initial weight (*W*_0_) and incubated in 50 mL DW (0.8% v/v) at 60 rpm and 37 °C for 168 h (with media refreshing at 1 and 24 h). Stability testing was performed by weighing hydrogel samples (*W*_s_) at different time points of 1, 2, 3, 4, 24, 48, 72, 96, and 168 h. Results and observations were based on the remaining weight of the samples, calculated by the equation used in swelling behavior studies (eq 1):

1 $$R_{w}=\frac{W_{s}-W_{0}}{W_{0}}\times 100\%$$

### 2.5 *In Vitro* Cytotoxicity Assay

#### 2.5.1 Cell Culture

The HDFs and Balb/c 3T3 cells were used to evaluate the effects of the different ε-PL/HA hydrogels on cell viability. HDF cells were cultured in DMEM/F12 supplemented with 10% FBS at 37 °C in a humidified 5% CO_2_ atmosphere. Balb/c 3T3 cells were cultured in DMEM with 10% FBS under the same conditions. Both cell types were subcultured at least twice prior to experiments.

#### 2.5.2 Indirect Cytotoxicity Assay on HDFs

Each hydrogel sample (200 mg) was washed three times with DPBS (pH 7.1–7.7; Sigma-Aldrich, cat. no. D1408) and allowed to swell for 4 h at 37 °C in DPBS. Afterward, the swollen hydrogel was cultured in DMEM for 24 h at 37 °C under 5% CO_2_ atmosphere. The culture media containing the released products from the hydrogel were collected and diluted with fresh growth medium using a dilution factor of 2.15 (200, 93.023, 43.27, 20.12, 9.36, 4.35, 2.025 mg/mL). The experimental approach was consistent with the OECD GD 129 standard procedure.^53^ HDF cells were seeded in 96-well plates at 1.75 × 10^3^ cells/100 μL/well (∼5500 cells/cm^2^) and allowed to reach 50–60% confluence before treatment. Hydrogel extracts were added and incubated for 48 h. One untreated plate was retained at *t* = 0 to normalize growth rates. Cell viability was assessed using LIVE/DEAD staining with Hoechst 33342 and propidium iodide (PI), followed by fixation in 4% paraformaldehyde. High-content imaging (InCell 2200, *GE HealthCare*, *USA*) was used to capture five fields/well with a 10× lens (DAPI (from 4′,6-diamidino-2-phenylindole) channel: Ex 390 nm/Em 432.5 nm, PI channel: Ex 542 nm/Em 597 nm). CellProfiler (v4.2.5) was used for automated nuclei segmentation and PI intensity quantification. Cells were classified as live (none to low PI) or dead (high PI), and viability values were normalized to negative control wells. Controls included the following: untreated cells (negative, 100% viability), Geneticin (G418, positive cytotoxic control), and medium-only controls. Each condition was tested in triplicate wells across three independent experiments (*n* = 3).

#### 2.5.3 Direct Cytotoxicity Assay on Balb/c 3T3 Cell Line

First, hydrogels were preswelled, as described in Section 2.5.2. Then, the swollen hydrogel samples were incubated with 1 mL DMEM/10%FBS (this volume of media does not cover the upper surface of the hydrogel). Then, 10 μL of Balb/c 3T3 (3 × 10^4^ cells/cm^2^) was pipetted on top of the hydrogel, and an additional 100 μL of cell culture media was added to fully cover the hydrogel. After 24 h, cell metabolic activity was measured using CellTiter Blue, according to the manufacturer’s recommendations.^54^ Fluorescence was measured using the Tecan plate reader (Männedorf, Switzerland), at 560Ex/590Em (*n* = 3 measurements per well). The resulting data were converted into cell viability (%) using eq 1:

2 $$Cell\;Viability,\%=\left(\frac{Average\;Fluorescence_{560Ex/590Em}-Negative\;control}{Positive\;control-Negative\;control}\right)\times 100$$

The morphology of the cells in contact with the hydrogel was assessed by using calcein staining. After 24 h of culture, 500 μL of 0.001 v/v% of calcein/DMEM solution was added to each well containing the hydrogel. After 20 min of incubation, cell morphology was evaluated using an OPTIKA ECO IM-5 fluorescent microscope (OPTIKA Srl, Italy). Experiments were performed in triplicate wells across three independent replicates.

### 2.6 Statistical Analysis

All results were expressed as the mean value ± standard deviation (SD) of at least three independent samples from ε-PL and each ε-PL/HA hydrogel series. One- or two-way analysis of variance (ANOVA) with Tukey’s multiple comparisons was used during the data analysis to determine statistical significance. Statistically significant results were considered as of *p* < 0.05 (ns – >0.05, * – <0.05, ** – <0.01, *** – <0.005, and **** – <0.001). Statistical analysis was performed by using IBM SPSS Statistics 23 software.

## 3 Results and Discussion

### 3.1 Rheological Studies

A temperature-dependent viscosity test was performed on all ε-PL/HA hydrogels to evaluate their thermoresponsive behavior and viscosity stability across a temperature range of 4 to 37 °C (Figure 2F). The viscosity of each hydrogel remained stable between 3500 and 5500 Pa·s across the tested temperature range (4–37 °C), with no statistically significant differences (*p*  > 0.05). This thermal stability supports hydrogel consistency at both room temperature (23 °C) and physiological temperature (37 °C), indicating their suitability for clinical use. The comparable viscosity profiles across all hydrogel series are attributed to the constant molar concentration of high molecular weight (HMW) HA (Table 1), which, through chain entanglement and hydrogen bonding, stabilizes the hydrogel network.^43,55^ As seen in Figure 2F, three distinct rheological regions were observed: Region I (4–15 °C), recognized as typical Newtonian behavior, with slight viscosity reduction as temperature increased; Region II (15–30 °C), where temperature-dependent behavior reverses upon the shear-dependent divergence point; and Region III (>30 °C), where a slight increase in viscosity could be observed, as from this point, the viscosity value changes as a function of the shear rate.^56^

**Figure 2:**
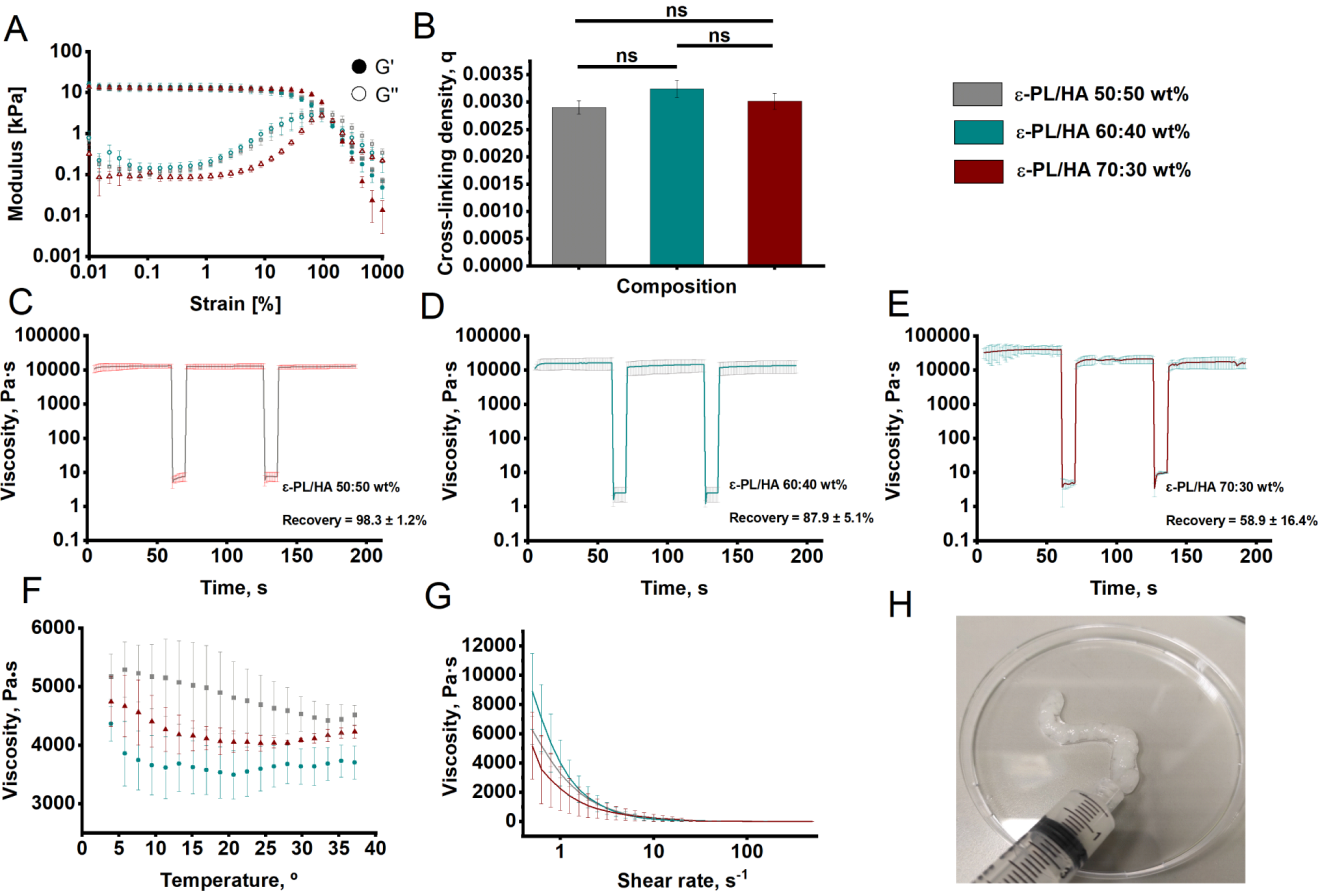
Rheological characterization of the ε-PL/HA hydrogels.ε-PL/HA 50:50 wt % colored as gray, 60:40 wt % -– dark green and 70:30 wt % -– dark red. **(A,B)** Amplitude sweep and cross-linking density. Amplitude sweep studies were obtained at a constant temperature of 25 °C and frequency of 1 Hz. Amplitude strain ranged from 0.01 to 1000%. **(C–E)** Recovery cycle tests done in flow mode under constant temperature of 25 °C, within 5 (3 + 2) cycles corresponding to 3*x*—shear rate at 0.1 s^–1^ for 60 s within LVR, and 2*x*—stress-induced cycles at 200 s^–1^ for 10 s; (**F**) Temperature-dependent viscosity test performed in flow mode at a constant frequency of 1 Hz, within temperature range from 4 to 37 °C with 1 °Cstep (*T* = 4 °C; ns for all hydrogel series: ε-PL/HA 50:50 wt %, ε-PL/HA 60:40 wt %, ε-PL/HA 70:30 wt %; *T* = 25 °C; ns for all hydrogel series; *T* = 37 °C; ns for all hydrogel series); (G) Shear rate dependent viscosity studies performed in flow mode under constant temperature of 25 °C within the range 0.1–500 s^–1^ shear rate with logarithmic step; **(H)** Illustration of the ε-PL/HA hydrogel sample extruded from the syringe with an inner diameter of 2.1 mm. Three replicates were used to ensure qualitative measurement results..

To further evaluate the flow behavior and injectability of the ε-PL/HA hydrogels, shear rate-dependent viscosity tests were performed (Figure 2G). These tests confirmed that all hydrogel series exhibit pronounced shear-thinning behavior, a key property for syringe-injectable biomaterials. The viscosity remained high at low shear rates (<1 s^–1^), particularly for the ε-PL/HA 50:50 wt %, suggesting a more entangled polymer network and stronger intermolecular interactions. In contrast, the ε-PL/HA 60:40 wt % and ε-PL/HA 70:30 wt % exhibited progressively lower viscosity, likely due to reduced cross-linked density and polymer interactions. All hydrogel formulations reached a viscosity plateau at higher shear rates (>10 s-1), indicating smooth syringeability and fluid-like behavior under injection-relevant conditions.^57^ The cyclic strain time sweep studies were performed to simulate the shear rate stress caused during extrusion from a syringe and to observe matrix recovery through viscosity values (Figure 2C–E). All ε-PL/HA hydrogel formulations exhibited rapid viscosity self-recovery within 15 s after high shear, indicating the ability to restore their structural state postdeformation.^58,59^ However, it was observed that ε-PL/HA 70:30 wt % hydrogels exhibited significantly reduced recovery capability, with a measured recovery rate of 58.9 ± 16.4%, compared to 98.3 ± 1.2% for ε-PL/HA 50:50 wt % and 87.9 ± 5.1% for ε-PL/HA 60:40 wt %, respectively. This significantly lower recovery in the 70:30 wt % hydrogel may be attributed to excess free, uncross-linked ε-PL chains, which can interfere with network cohesion and hinder viscosity restoration. Similar effects have been reported in other polymer systems, such as CMC-based hydrogels, where free polymer chains negatively impact flow and recovery behavior.^60^

Further insights were obtained from amplitude sweep tests (Figure 2A) and cross-linking density calculations (Figure 2B), which showed no significant differences among the hydrogel compositions. Besides the viscoelastic behavior described in our previous work,^42,43^ extracted storage modulus (G′) values were used to calculate the cross-linking density of prepared ε-PL/HA hydrogels (Figure 2A,B). The cross-linking density (*q*) was obtained as 0.0029 ± 0.00012, 0.0032 ± 0.00015, and 0.003 ± 0.00014 mol·m^–3^ for ε-PL/HA hydrogels with 50:50, 60:40, and 70:30 wt %, respectively. A nonsignificant difference was found between cross-linking density values within all hydrogel series, suggesting that ε-PL and HA cross-linked equally. To sum up, these uncross-linked ε-PL chains were introduced during the fabrication process, where all ε-PL/HA hydrogel series were prepared with a constant EDC/NHS cross-linker concentration, while varying the ε-PL to HA mass ratios.

Overall, rheological characterization confirmed that ε-PL/HA hydrogels possess key properties for syringe-based delivery, including shear-thinning behavior, rapid self-recovery, temperature stability, and tunable viscosity. These attributes ensure consistent performance before and after injection and support the potential of the hydrogels for clinical applications, such as wound infection treatment and 3D bioprinting.

To support the rheological data, additional indicators of cross-linking efficiency were analyzed, including the NH_3_^+^/NH_2_ ratio,^42,43^ gel fraction,^42,43^ swelling capacity, cross-linking density, and gelation time. These results are summarized in Table 2. With increasing ε-PL content, FTIR analysis showed a shift in the amide bands and an increase in the NH_3_^+/^NH_2_ absorbance ratio, reflecting more free ε-PL residues at higher ε-PL mass ratios. This trend is consistent with the rheological results, confirming that higher ε-PL content leads to less dense networks with higher swelling capacity.

**Table 2:** Crosslinking Characteristics of ε-PL/HA Hydrogels

Designation	NH_3_^+^/NH_2_ratio (I_3062_/I_3228_)^42,43^	Gel fraction, % ^42,43^	Swelling capacity, %^a^	Cross-linking density (*q*, mol·m^–3^)	Gelation time, min^b^
**ε-PL/HA 50:50 wt %**	0.73	52 ± 0.05	166.07± 34.8	0.003 ± 0.0001	34
**ε-PL/HA 60:40 wt %**	0.85	56.2 ± 0.2	277.2 ± 13.6	0.003 ± 0.0001	41
**ε-PL/HA 70:30 wt %**	0.83	54.7 ± 1.6	441.5 ± 41.8	0.003 ± 0.0001	46

a See Figure S2.

b See Figure S1.

### 3.2 Minimum Inhibitory/Bactericidal Concentration of ε-PL

The antibacterial activity of pure ε-PL was evaluated by determining the MIC and MBC values against both the Gram-positive and Gram-negative bacterial species, including multidrug-resistant isolates. This property is particularly important, as the bacterial strains selected for this study are among the most common pathogens associated with postsurgical, skin, oral, and implant-related infections.^61,62^ The MIC/MBC experiments (Figure 3A) demonstrated the potent inhibitory capability of ε-PL against a wide range of bacterial species. The results revealed that ε-PL at a concentration of 100 μg/mL effectively inhibited the growth of both Gram-negative and Gram-positive bacteria, including multidrug-resistant strains. Remarkably, a lower concentration of ε-PL, such as 75 μg/mL, was sufficient to achieve a bactericidal effect against *E. coli*, *ESBL* *E. coli*, *S. epidermidis*, *S. aureus*, and *MRSA*.

**Figure 3:**
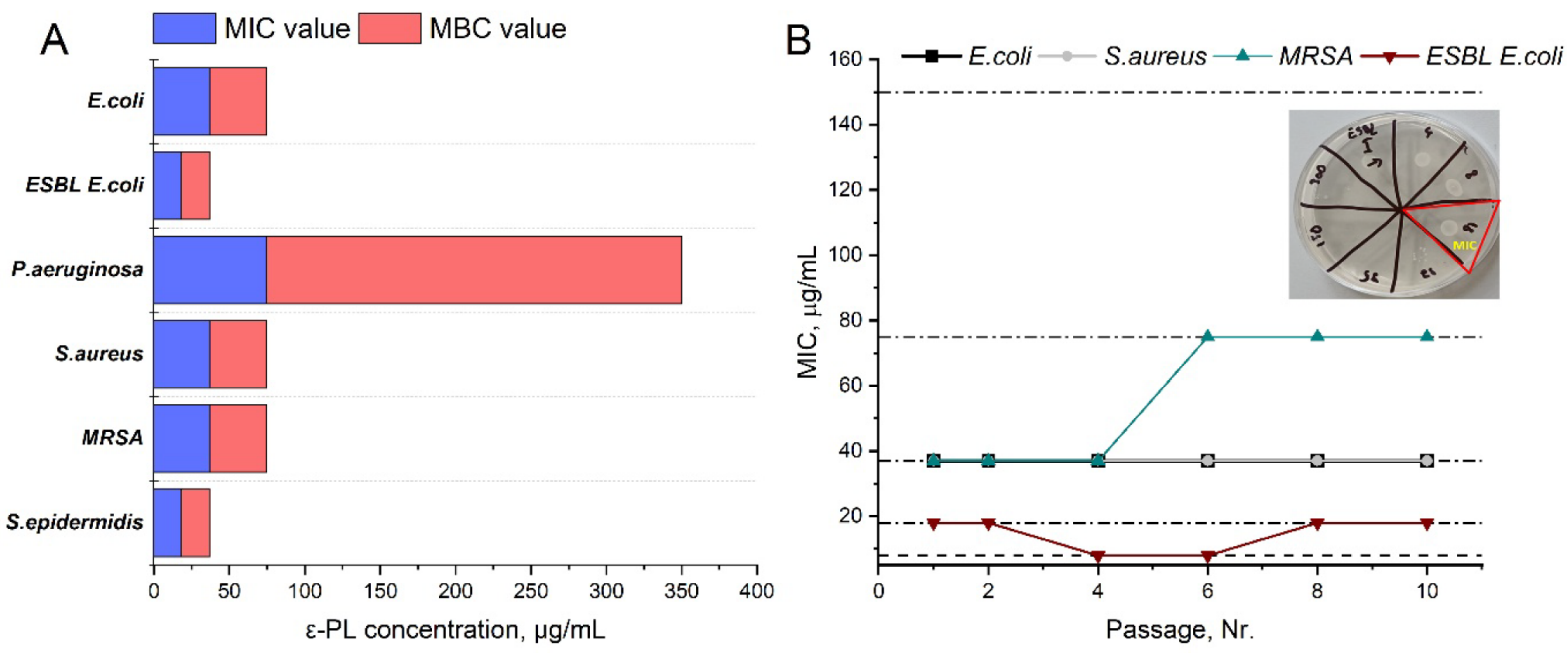
Antibacterial activity of pure ε-PL. **(A)** Minimum inhibitory/bactericidal concentration (MIC/MBC) studies against different bacterial species: *E. coli*, *P. aeruginosa*, ESBL *E. coli*, *S. aureus*, *S. epidermidis*, and MRSA (blue bars indicate obtained MIC values and red bars – MBC values); **(B)** Resistance development studies against *S. aureus*, MRSA, *E. coli* and ESBL *E. coli* within 10 passages and plotted curves reveal changes of MIC value within 1, 2, 4, 6, and 10 passages.

However, for Gram-negative *P. aeruginosa*, significantly higher concentrations of ε-PL (∼350 μg/mL) were required to achieve a bactericidal effect. This observation may be attributed to the unique structural and functional characteristics of *P. aeruginosa* compared to other representatives of the Gram-negative family (e.g., *E. coli*), including its robust outer membrane, multiple efflux pumps, and highly adaptive gene expression system.^63^ These characteristics and well-developed resistance mechanisms contribute to its classification as a high-risk opportunistic pathogen in clinical settings.^63^ In addition to the general structural barriers common to Gram-negative bacteria, several studies have explored how these intrinsic and acquired features interfere with the mechanisms of action of even the most potent antibiotics.^63^ Computational studies have shown that divalent cations and their interaction with anionic lipopolysaccharides (LPS) increase the stiffness of the outer membrane, enhancing membrane integrity and reducing the cell surface anionicity.^63^ This structural rigidity is a critical factor that may significantly hinder the antibacterial action of ε-PL, as previously described.

### 3.3 Evaluation of Bacterial Resistance Development against ε-PL

For the first time, a comprehensive evaluation was conducted to assess the potential of the antimicrobial polypeptide ε-PL to induce bacterial resistance, including in multidrug-resistant isolates. The MIC values were measured after passages in the presence of a range of ε-PL concentrations to track the resistance development. Bacterial suspensions were inoculated with ε-PL (8, 18, 37, 75, and 150 μg/mL) and incubated for 24 h at 37 °C on TSA plates (illustration provided in Figure 3B). The results (Figure 3B) demonstrated that *E. coli* and *S. aureus* remained consistently sensitive to ε-PL, as the MIC values did not change compared with initial results (Figure 3A). Similarly, the MIC value for *ESBL* *E. coli* remained stable at 18 μg/mL over 10 passages, indicating no resistance development. A slight MIC increase was observed for MRSA after 10 passages. However, this shift was within one 2-fold dilution, which corresponds to the expected biological and technical variability of MIC assays,^64^ and therefore does not indicate actual resistance development. Tan et al. previously described the inhibition mechanism of ε-PL against *S. aureus* as a dual-action process: membrane disruption via the classical carpet-like model and participation in the tricarboxylic acid cycle, affecting aconitase and succinate dehydrogenase enzymes.^65^ It is hypothesized that the minor MIC fluctuation observed for MRSA may be due to suppression of the secondary metabolic mechanism of ε-PL, thereby requiring slightly higher concentrations to maintain inhibitory efficacy.

### 3.4 Direct Studies of Antibacterial Activity of Prepared Hydrogels

The bactericidal activity of the ε-PL/HA hydrogels with ε-PL:HA mass ratios of 50:50, 60:40, and 70:30 wt % was evaluated using a direct contact test (Figure 4). All tested hydrogels demonstrated statistically significant (*p* < 0.05) antibacterial activity, expressed as log reduction in CFU compared to the untreated control. Complete eradication of *S. epidermidis* was achieved by all hydrogel series, while significant log reductions were also observed for *S. aureus*, *E. coli*, *P. aeruginosa* *, MRSA,* and *ESBL* *E. coli*. These findings are consistent with the high sensitivity observed in MIC/MBC tests of pure ε-PL. The bactericidal effect was most pronounced in hydrogels with a higher ε-PL content, reflecting the increased availability of free, primary ε-amino (−NH_2_) groups that become protonated [NH_3_^+^] in aqueous conditions, contributing to improved antibacterial activity (Figure 4). These positively charged groups enable electrostatic interactions with negatively charged bacterial membranes, enhancing bactericidal efficacy. As shown in Figure 1B, the cross-linking density (*q*, mol·m^–3^) remained consistent across all hydrogel series, indicating that differences in antibacterial performance were related to the number of free, uncross-linked ε-amino (−NH_2_) groups rather than the cross-linking degree. Our previous reports^42,43^ confirmed this relationship, showing that increasing ε-PL mass ratios led to higher NH_3_^+^/NH_2_ values (0.73, 0.85, and 0.83 for ε-PL/HA 50:50, 60:40, and 70:30 wt %, respectively), consistent with FTIR analysis. These data demonstrate that a higher ε-PL content correlates with a greater concentration of positively charged NH_3_^+^ groups, critical for antibacterial efficacy. Polymers containing positively charged functional groups, such as amines, are known to disrupt bacterial membranes via electrostatic attraction, particularly when cationic charge reaches a critical threshold (multivalence effect).^66^ In line with the MIC/MBC results, all hydrogels also demonstrated statistically significant inhibition of *P. aeruginosa* growth. However, complete eradication was observed only for the ε-PL/HA 70:30 wt % hydrogel containing the highest free NH_3_^+^ content. For the other isolates (*S. aureus*, *E. coli*, *MRSA, ESBL* *E. coli*), log reduction was comparable across the hydrogel series, suggesting that hydrogel composition primarily influences *P. aeruginosa* susceptibility. These findings indicate that a sufficient concentration of free, uncross-linked primary ε-amino (−NH_2_) groups is essential for effective electrostatic interactions, which promote bacterial membrane disruption and may interfere with the metabolic pathways, thereby enhancing bactericidal efficacy.

**Figure 4:**
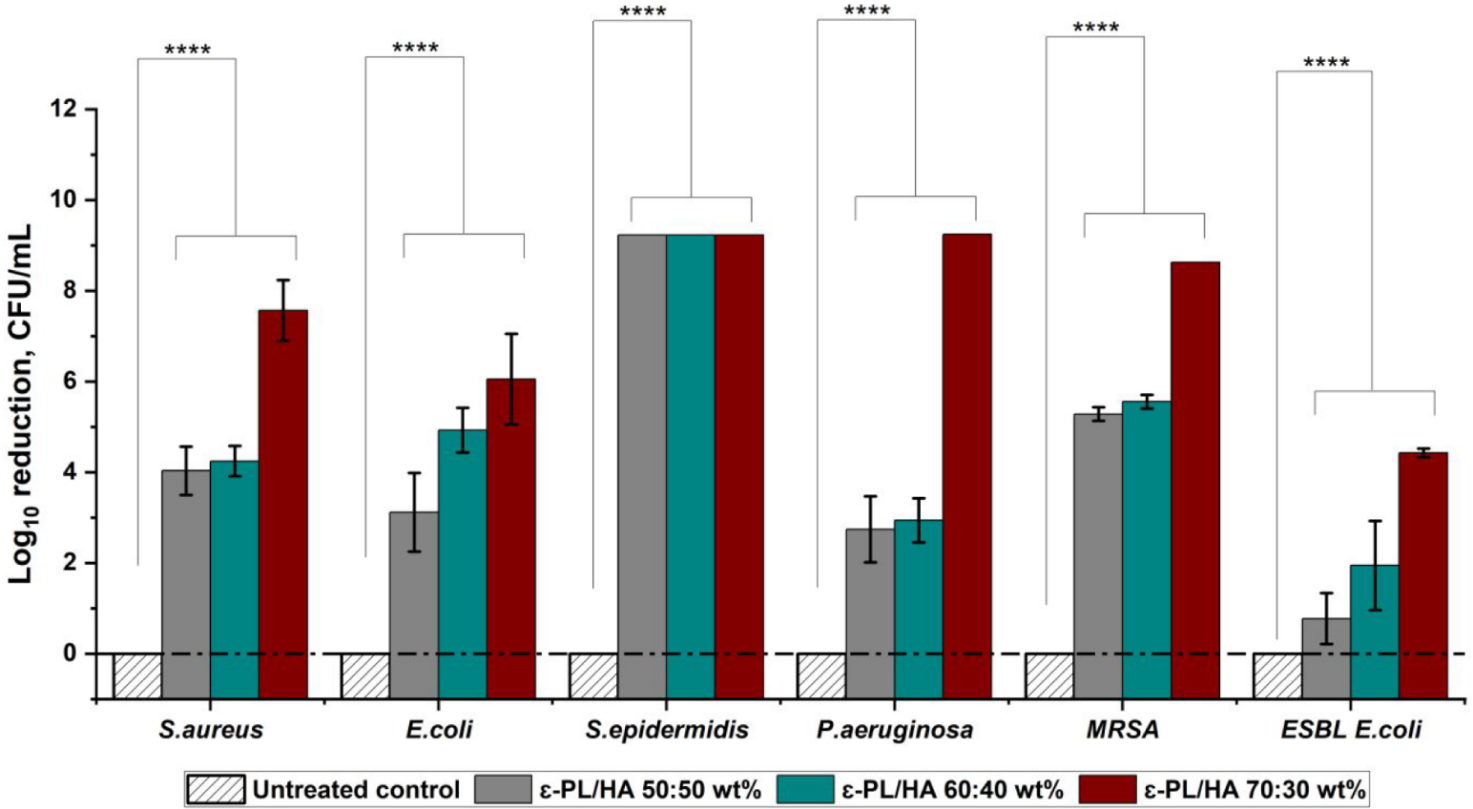
Direct antibacterial activity of ε-PL/HA hydrogels against *S. aureus*, *E. coli*, *S. epidermidis*, *P. aeruginosa*, *MRSA*, and *ESBL* *E. coli* after 24 h. Data are expressed as log reduction in CFU (mean ± SD, *n* = 3), and statistical analysis was performed by one-way ANOVA: ns – >0.05, * – <0.05, ** – <0.01, *** – <0.005, and **** – <0.001. Dash-dotted lines indicate compositions where complete eradication was achieved.

### 3.5 Biofilm Biomass Quantification via Crystal Violet Assay

The antibiofilm activity of ε-PL/HA hydrogels was evaluated using the conventional crystal violet assay to comprehensively assess their antibacterial performance. The activity was tested against two clinically relevant bacterial strains, *S. aureus* and *MRSA*, which are frequently associated with healthcare- and medical device-related biofilm infections. Bacterial biofilms are complex microbial communities encased in extracellular polymeric substances, representing a significant challenge in treating infections and contributing significantly to their persistence.^49^ The ability of ε-PL/HA hydrogels to inhibit or reduce biofilms would further strengthen their antibacterial performance and highlight their potential biomedical applications. This is particularly important as conventional antibiotics often display reduced efficacy against biofilms due to several factors, including: (i) metabolic alterations of bacteria within the biofilm, (ii) limited penetration of antibiotics through the extracellular matrix, (iii) inactivation of antibiotics by matrix components, (iv) inoculum effects related to the high bacterial density, and (v) enhanced horizontal transfer of resistance mechanisms due to close cell-to-cell proximity.^51^ Results on the antibiofilm activity of ε-PL/HA hydrogels are shown in Figure 5. The obtained results revealed that all ε-PL/HA hydrogel compositions achieved a statistically significant reduction (*p* < 0.001) in biofilm biomass formed by *S. aureus* and *MRSA* within 24 h compared to untreated controls (Figure 5). For the treated control, a vancomycin/gentamicin mixture was used. This choice was based on literature evidence identifying vancomycin as a first-line clinical antibiotic against *S. aureus*, including *MRSA* infections, and demonstrating the synergistic activity of vancomycin–gentamicin combinations against *S. aureus* biofilms.^67,68^ Consistent with these reports, the applied antibiotic mixture (3 mg/mL vancomycin and 2 mg/mL gentamicin) also produced a significant reduction (*p* < 0.05) in established biofilms of both bacterial strains. No significant difference was observed between the antibiotic mixture and the 50:50 wt % hydrogel composition, indicating that this formulation was equally effective as clinically used antibiotics. However, the 60:40 and 70:30 wt % hydrogel formulations showed significantly greater biofilm reduction (*p* < 0.05) than the treated control for both *S. aureus* and *MRSA*. These findings demonstrate the strong antibiofilm properties of ε-PL/HA hydrogels and underline the influence of increasing ε-PL content on their antibacterial efficacy.

**Figure 5:**
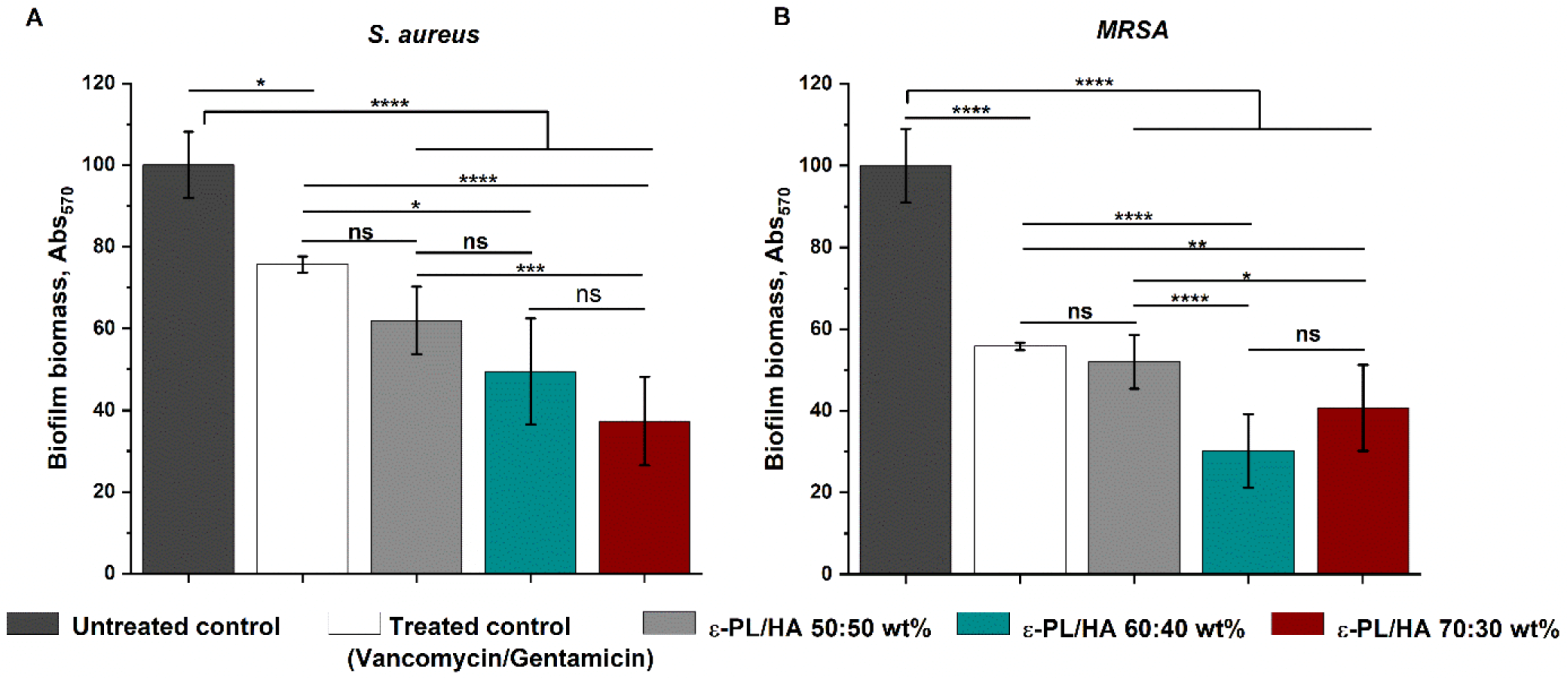
Antibiofilm activity of ε-PL/HA hydrogels was tested by crystal violet biofilm biomass quantification testing against (A) *S. aureus* and (B) *MRSA* bacteria. Untreated controls consisted of a 1:50 diluted overnight bacterial suspension in TSB. A vancomycin (3 mg/mL) and gentamicin (2 mg/mL) mixture served as the antibiotic control. Each group was tested in quadruplicate (*n* = 4). Biofilm biomass was quantified by absorbance at 570 nm. Data are presented as mean ± SD, and statistical analysis was performed by one-way ANOVA: ns – >0.05, * – <0.05, ** – <0.01, *** – <0.005, and **** – <0.001.

### 3.6 Antibiofilm Activity of ε-PL/HA Hydrogels via Live/Dead and Viability Assay

Live/Dead staining and viability assays against *S. aureus* supported previous findings. After 24 h, fewer bacteria were able to attach to the surface of the prepared hydrogels and form biofilms compared to Ti discs, as observed in the Live/Dead images (Figure 6). Quantitative analysis of viable *S. aureus* bacteria confirmed a significant reduction (*p* < 0.05) in bacterial colonies across all ε-PL/HA hydrogel compositions compared to the initial concentration (*t* = 0, Figure 6).

**Figure 6:**
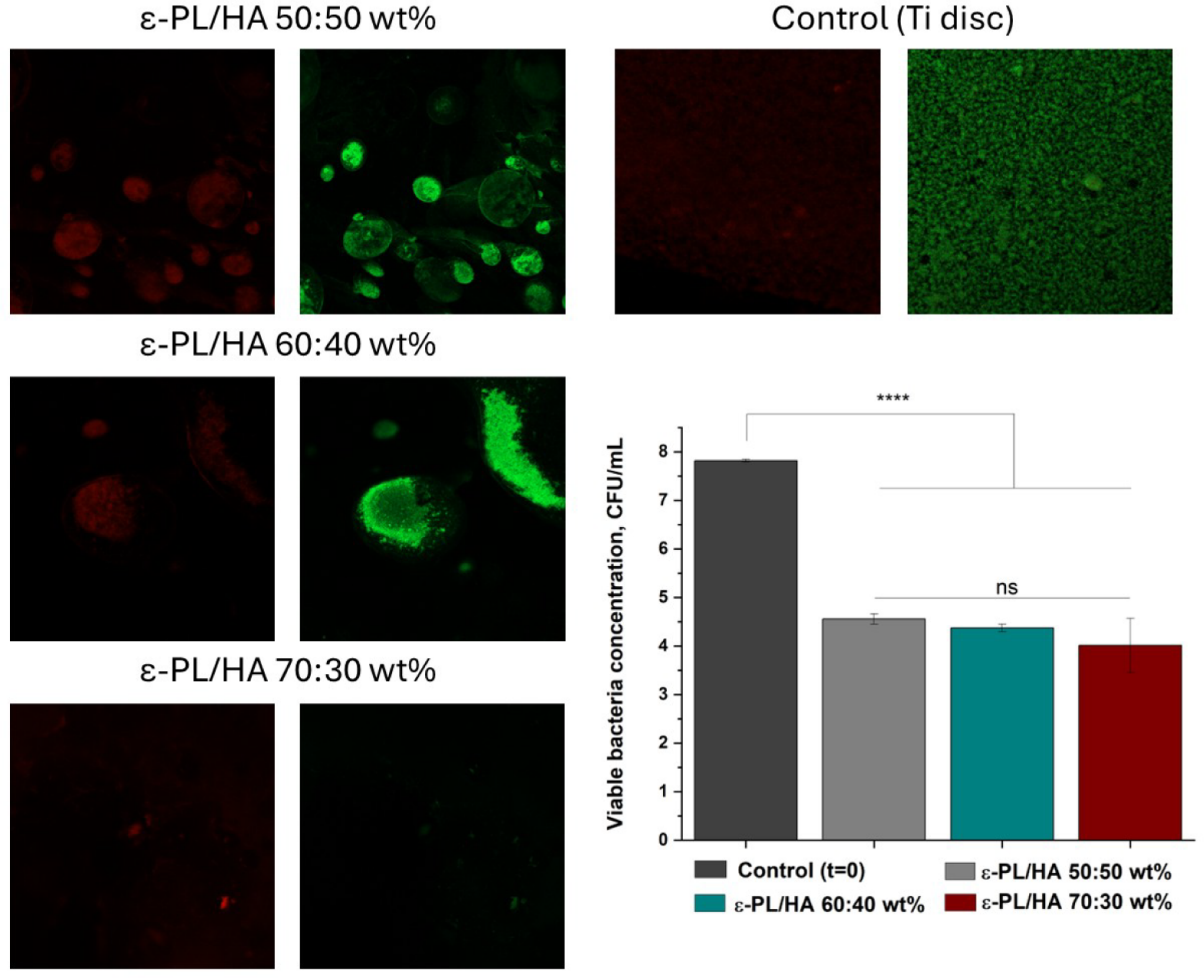
Live/Dead and viability assay for ε-PL/HA hydrogels against *S. aureus*. Live/Dead microscopical images (scale 200 μm) illustrating bacteria attaching after 24 h of incubation on hydrogel surface. Red fluorescent dye represents dead bacteria, while Green dye represents viable bacteria found on the hydrogel surface. Quantitative graph represents viable bacteria concentration after 24 h compared with initial bacteria suspension concentration. Data are expressed as log viable bacteria in CFU (mean ± SD, *n* = 3). Statistical analysis was performed by one-way ANOVA: ns – >0.05, * – <0.05, ** – <0.01, *** – <0.005 and **** – <0.001.

In contrast, after 72 h, Live/Dead staining revealed a markedly higher presence of *S. aureus* (Figure S3), which was further confirmed by increased viable bacterial concentrations (Figure S3). These results indicate that while ε-PL/HA hydrogels effectively reduce initial bacterial attachment and early biofilm formation, their antibiofilm activity is less pronounced against mature biofilms over longer incubation periods.

### 3.7 Long-Term Indirect and Direct Antibacterial Potential of ε-PL/HA Hydrogels

The sustained antibacterial performance of ε-PL/HA hydrogels (50:50, 60:40, and 70:30 wt %) was assessed over 168 h (7 days) using two complementary approaches: an indirect test (supernatants collected) to measure antibacterial activity mediated by released, free ε-amino groups of ε-PL (Figure 7A), and a direct contact test to evaluate bactericidal effects during prolonged exposure to the hydrogel surface (Figure 7B). Antibacterial efficacy was expressed as a log reduction in CFU compared to the untreated control. In the indirect test, the 70:30 wt % hydrogel achieved the most significant log reduction in CFU for both *S. aureus* and *E. coli*, followed closely by the 60:40 wt % formulation. The 50:50 wt % hydrogel produced only moderate inhibition, with no statistically significant difference compared to higher ε-PL ratio hydrogels.

**Figure 7:**
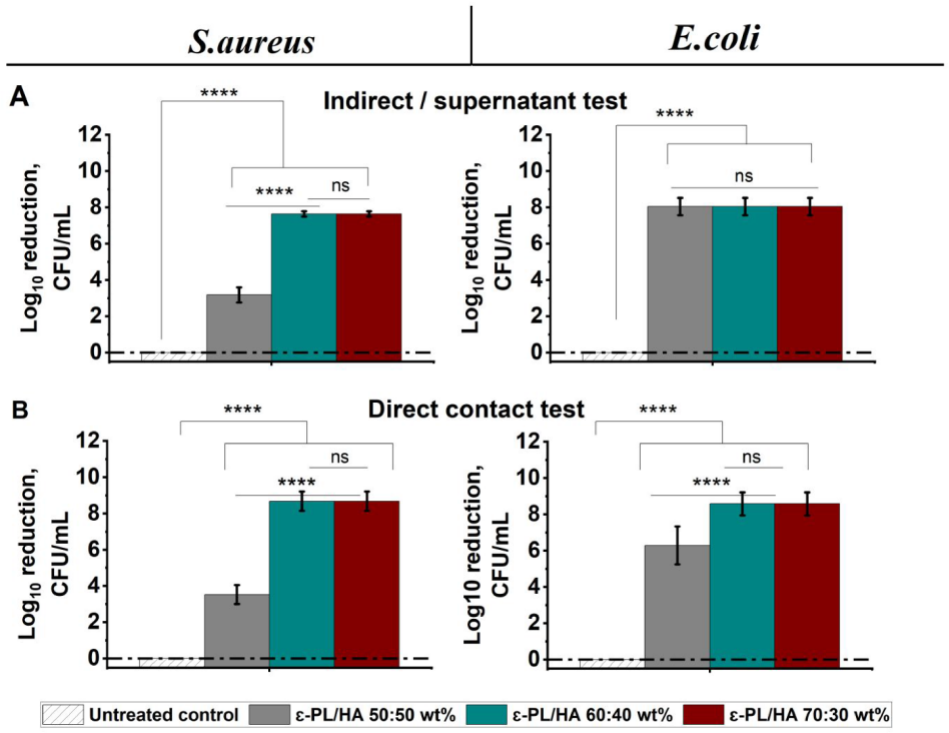
Long-term antibacterial activity of ε-PL/HA hydrogels against *S. aureus* and *E. coli*. (A) Indirect antibacterial test (supernatants collected after 168 h); (B) Direct antibacterial activity after 168 h direct contact with hydrogels. Data are expressed as log reduction in CFU (mean ± SD, *n* = 3). Statistical analysis was performed by one-way ANOVA: ns – >0.05, * – <0.05, ** – <0.01, *** – <0.005, and **** – < 0.001. Dash-dotted lines indicate compositions where complete eradication was achieved.

In the direct contact test, all hydrogels maintained antibacterial activity over 168 h, but the 60:40 and 70:30 wt % hydrogels were markedly more effective, achieving complete eradication of *S. aureus* and a substantial log reduction in *E. coli* CFU. In contrast, the 50:50 wt % hydrogel exhibited lower bactericidal efficacy.

These findings indicate that a higher ε-PL content increases the availability of free, uncross-linked ε-amino (−NH_2_) groups, which sustain electrostatic interactions with bacterial membranes over time. The presence of free NH_3_^+^ groups appears crucial for maintaining the multivalence effect, particularly against Gram-negative bacteria such as *E. coli*. However, excessive cross-linking can reduce the number of free NH_3_^+^ groups, potentially compromising antibacterial action. Overall, the results highlight the importance of balancing cross-linking density with the availability of functional groups to achieve a long-term antibacterial performance. While the present study confirms sustained bactericidal potential for at least 7 days, extended evaluations (≥5 weeks) are needed to verify durability. Previous reports by A. Smola-Dmochowska et al. with *P. aeruginosa* support that maintaining high local concentrations of antibacterial molecules is essential for sustained efficacy.^66^ Notably, our earlier work by K. Salma-Ancane et al. also revealed that increasing the ε-PL ratio improves antibacterial potency but may reduce mammalian cell viability,^42^ underscoring the need to optimize formulations for both efficacy and cytocompatibility.

### 3.8 Swelling Behavior and Structural Stability Studies of ε-PL/HA Hydrogels

Stability studies were performed on ε-PL/HA hydrogels to investigate structural changes during incubation under dynamic conditions for 168 h. This could also help interpret results from the long-term antibacterial activity studies (Section 3.7). As described in Section 3.7, hydrogels and supernatants showed significant bacterial colony reduction compared to controls. The stability test results are shown in Figure S2 as the remaining hydrogel weight over 168 h, with measurements at 1, 2, 3, 4, 24, 48, 72, 96, and 168 h.

Within the first 24 h, the swelling curves of the prepared ε-PL/HA hydrogels with different compositions closely reproduced the swelling behavior observed in our previous study.^43^ In the first hour, the hydrogels rapidly absorbed water, followed by network reorganization, reaching swelling capacities of 175%, 280%, and 440% for 50:50, 60:40, and 70:30 wt % compositions, respectively. After this initial phase, the hydrogel weight remained stable, indicating that the hydrogels did not degrade within this time frame. These results agree with our recent study by Rubina et al., where ε-PL/HA hydrogels maintained their weight in PBS containing hyaluronidase for up to 5 weeks, followed by gradual weight loss and complete degradation by 20 weeks.^44^

Based on our stability studies and previously reported enzymatic degradation profiles, it can be concluded that the hydrogels are stable for at least 168 h under dynamic conditions in both water and physiological-like environments. These findings and the long-term antibacterial studies indicate that although the bulk degradation was not detectable, trace releases of uncross-linked ε-PL occurred at levels sufficient to sustain antibacterial activity in the hydrogel and its supernatant up to 168 h.

### 3.9 Cell Viability Assay

The indirect cytotoxicity of ε-PL/HA hydrogels was first assessed on HDFs using hydrogel extracts after 48 h of exposure (Figure 8A). Cell viability remained above 70% up to 4.35 mg/mL extract concentration for all hydrogel compositions, indicating no cytotoxicity. Notably, at the lowest concentration of 2.025 mg/mL, the ε-PL/HA 50:50 wt % hydrogels enhanced HDF viability compared to the controls, suggesting that low concentrations of ε-PL may support cell viability. This observation aligns with previous findings by Tan et al., where low doses of ε-PL were shown to increase intracellular amino acid levels, indicating a self-protective mechanism against environmental stressors.^65^ This effect may arise from metabolic modulation rather than cytotoxicity, implying that careful dosing can exploit the beneficial properties of ε-PL while minimizing cytotoxic effects. Therefore, at lower concentrations, ε-PL may promote cell survival, particularly in designing highly biocompatible hydrogels. At higher concentrations, differences between compositions became evident: the ε-PL/HA 50:50 wt % maintained cell viability >70% up to 93.023 mg/mL, while the ε-PL/HA 60:40 wt % showed cytotoxic effects starting from 20.12 mg/mL, and the ε-PL/HA 70:30 wt % exhibited significant cytotoxicity at 9.36 mg/mL. All hydrogel series exhibited significant cytotoxicity at the highest tested concentration (≥93.023 mg/mL). Among the tested series, ε-PL/HA 50:50 wt % hydrogels displayed the most favorable balance between maintaining cell viability and minimizing cytotoxic effects. Direct cytotoxicity testing on Balb/c 3T3 cells (Figure 8B) revealed that all hydrogel series exhibited mild cytotoxicity, with cell viability decreasing to approximately 70% compared to the untreated control (*p* < 0.05). No significant differences were observed between hydrogel series (ns), indicating that the type of hydrogel series did not significantly influence cell viability. Comparing both results, it could be observed that HDF cells exposed to hydrogel extracts exhibited higher viability compared to Balb/c 3T3 cells directly cultured on the hydrogels. This is a common observation previously noticed in several studies.^42,69^ The difference in results directly relies on the testing approach, as indirect exposure limits the contact of cells with the hydrogel network. In contrast, a direct culture imposes both chemical and physical stress.

**Figure 8:**
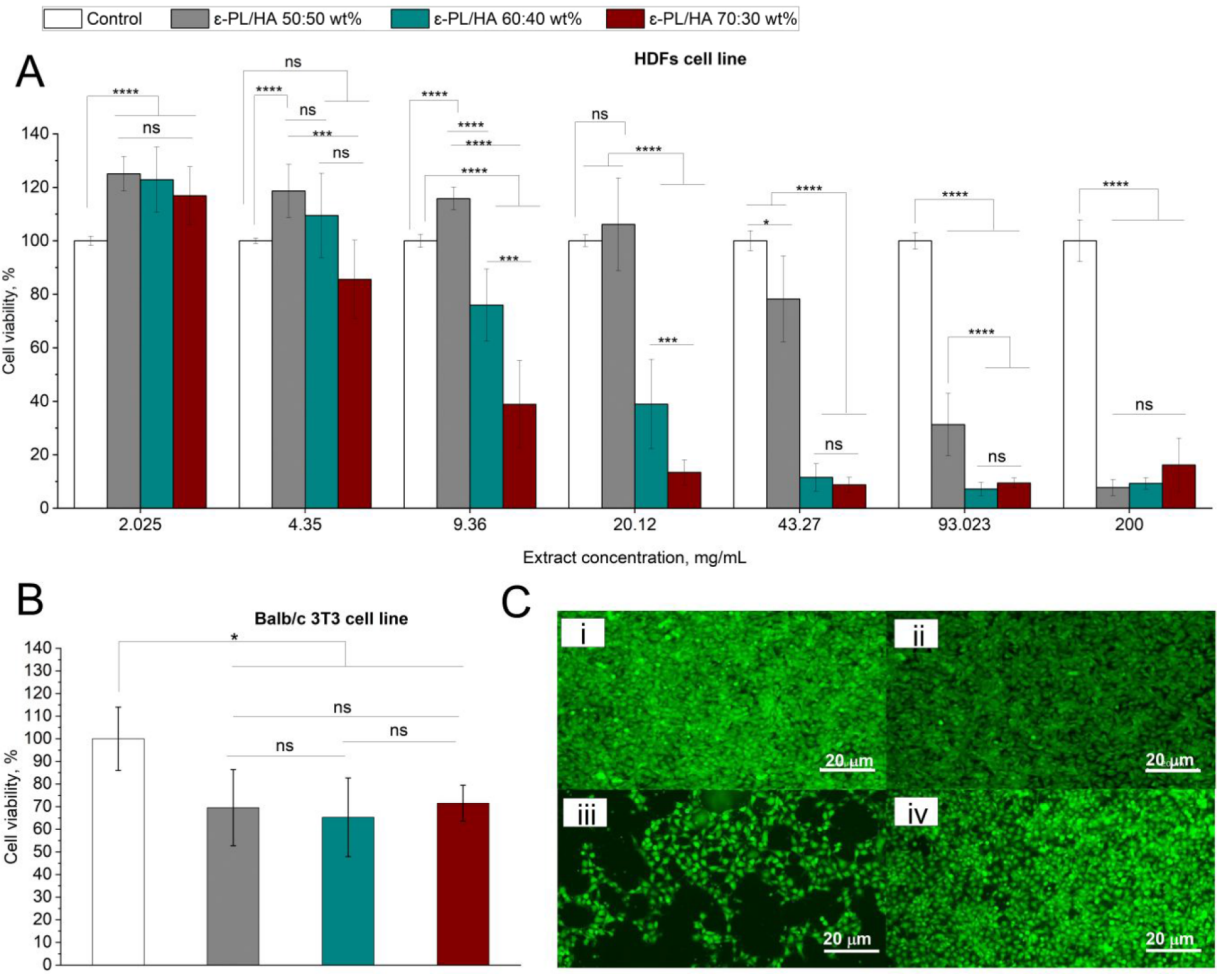
Cytotoxicity assay of ε-PL/HA hydrogels on HDFs and Balb/c 3T3 cells via indirect and direct tests. (A) Indirect assay on HDFs exposed to hydrogel extracts at concentrations ranging from 2.025 to 200 mg/mL for 48 h; (B) Direct assay on Balb/c 3T3 fibroblasts cultured on ε-PL/HA hydrogels (50:50, 60:40, 70:30 wt %) for 24 h; (C) Representative microscopic images of Balb/c 3T3 cells after 24 h direct culture: (i), (ii) cells in contact with ε-PL/HA 50:50 wt % hydrogel; (iii) negative control; (iv) positive control. Data are represented as mean ± SD (*n* = 3). Statistical analysis was performed by one-way ANOVA: ns >0.05, * <0.05, ** <0.01, *** <0.005, **** <0.001.

Representative microscopy images of Balb/c 3T3 cells after direct contact with hydrogels (Figure 8C, i–iv) confirmed that the cells maintained normal morphology and attachment, comparable to the negative control, whereas the positive control showed cell death. This suggests that although viability decreased slightly, overall cell integrity and morphology remained unaffected. Furthermore, the observed cytotoxicity of the hydrogels can be attributed to the antibacterial activity of ε-PL, which disrupts bacterial membranes but may also interact with mammalian cell membranes. This is consistent with the mechanism of polycationic hydrogels, which can induce electrostatic interactions with negatively charged cell membranes. However, by microscopic evaluation, cells grew and showed normal morphology when cultivated directly with the ε-PL/HA hydrogels. In the context of developing antibacterial hydrogels for tissue engineering, achieving a balance between antimicrobial efficacy and cytocompatibility is crucial.^70,71^ Although increasing the ε-PL content enhances bactericidal activity, it also raises the risk of cytotoxicity, underscoring the need for careful composition optimization. This balance often depends on both the concentration and the type of antimicrobial agents incorporated into the hydrogel network.^72^ These findings indicate that while a higher ε-PL content enhances antibacterial action, achieving a formulation with optimal cytocompatibility is essential for practical biomedical applications. The ε-PL/HA 50:50 wt % hydrogel demonstrated the most favorable balance, making it a promising candidate for tissue engineering.

## 4 Conclusions

This study demonstrated the development and evaluation of *in situ* forming, covalently cross-linked ε-polylysine/hyaluronic acid hydrogels with tunable ε-PL content (50:50, 60:40, and 70:30 wt %) for minimally invasive, syringe-based delivery to infection sites. Rheological characterization confirmed that the hydrogels exhibit favorable mechanical properties, including shear-thinning behavior, self-recovery capability, and consistent injectability across a physiologically relevant temperature range, supporting their practical use in clinical settings. *In vitro,* antibacterial assays revealed that the hydrogels provided intense bactericidal activity against a broad panel of clinically relevant Gram-positive and Gram-negative bacteria, including multidrug-resistant bacterial strains. They also demonstrated strong antibiofilm activity within 24 h and retained prolonged antibacterial efficacy, while ε-PL contributed intrinsic antibacterial activity without promoting bacterial resistance development. Cytocompatibility assessments showed a dose-dependent response of fibroblasts to the ε-PL concentration included in the hydrogels, highlighting the importance of ε-PL concentration in modulating cell viability. This study presents the first comprehensive evaluation of injectable ε-PL/HA hydrogels that exhibit favorable rheological features (shear-thinning behavior, injectability, self-recovery), sustained antibacterial efficacy, and biocompatibility. Combining the membrane-disruptive action of ε-PL with the biologically active properties of HA, these hydrogels represent a promising nonantibiotic strategy for localized treatment of bacterial wound infections, including those caused by antibiotic-resistant and biofilm-associated pathogens.

## Supplementary Material



## Notes

S.1.1. Time sweep studies: results and discussion, as well as gelation time values summarized in Table 2. Figure S1 within Section S.1.1. supporting Section 3.1. Figures S2 supporting Section 3.8. Figure S3 supporting Section 3.6 (PDF)
