# Supplementary material for: Injectable ε‑Polylysine/Hyaluronic Acid Hydrogels with Resistance-Preventing Antibacterial Activity for Treating Wound Infections
Source: ACS Appl Bio Mater. 2025 Oct 30;8(11):9916–30. doi: 10.1021/acsabm.5c01252 (PMC12628331; doi:10.1021/acsabm.5c01252)
Supplement: Supplementary file 1 [file mt5c01252_si_001.pdf]

Injectable ε-polylysine/hyaluronic acid hydrogels with resistance-preventing antibacterial activity for treating wound infections – Supporting Information

Artemijs Sceglovs[1,2], Claudia Siverino[3], Ingus Skadins[4], Marika Sceglova[1,2], Valdis Pirsko[5], Thomas Fintan Moriarty[3], Juta Kroica[4], Kristine Salma-Ancane[1,2*]

[1] *Institute of Biomaterials and Bioengineering, Faculty of Natural Sciences and Technology, Riga Technical University, Paula Valdena St. 3, Riga, LV-1048, Latvia*

[2] *Baltic Biomaterials Centre of Excellence, Headquarters at Riga Technical University, Paula Valdena St. 3, k-1, Riga, LV-1048, Latvia*

[3] *AO Research Institute Davos, Clavadelerstrasse 8, Davos 7270, Switzerland*

[4] *Department of Biology and Microbiology, Riga Stradins University, Dzirciema St. 16, Riga, LV-1007, Latvia*

[5] *Institute of Microbiology and Virology, Riga Stradins University, Ratsupites St. 5, Riga, LV-1067, Latvia*

* Corresponding author – Kristine Salma-Ancane, Riga Technical University, Paula Valdena St. 3, k-1, Riga, LV-1048, Latvia; kristine.salma-ancane@rtu.lv

Supplementary results

S.1.1. Time sweep studies

Oscillation Oscillatory rheology time sweep experiments were performed to determine the gelation time of ε-PL/HA hydrogels. Gelation time is a critical parameter for injectable biomaterials. In principle, time sweep curves display the storage modulus (G′) evolution over the experimental period. During gelation, the system transitions from a viscous-like state, characteristic of the synthesis precursors, into a soft, solid-like material. This transformation is reflected by the change in the balance between loss modulus and storage modulus, i.e., from G″ > G′ (viscous dominance) to G′ > G″ (elastic dominance). The crossover point (G′ = G″) is commonly interpreted as the gelation time [1].

Representative time sweep curves are shown in Figure S1. As noted in earlier work by Rubina et al. [1], sodium hyaluronate (pre-dissolved in distilled water) was used as the HA source. Since sodium hyaluronate behaves as a soft, solid-like material [2], the storage modulus (G′) exceeded the loss modulus (G″) from the start of the experiment. In the obtained curves (Figure S1B), G′ increased rapidly within the first 20 minutes (blue region), indicating the progression of chemical crosslinking within the reaction mixture. In parallel, axial force also rose sharply (Figure S1A), reflecting increased stiffness as the hydrogel pushed back against the geometry plate.

After approximately 15 minutes, axial force began to decline, whereas the storage modulus continued to rise until reaching its maximum at t = 20 min. The sharp decrease in axial force (Figure S1A) likely reflects hydrogel matrix rearrangement and reorganization, accompanied by sample shrinkage and syneresis (partial water expulsion). During this stage (red region), the gel network was still undergoing relaxation, leading to fluctuations and a gradual decrease in G′ despite ongoing crosslinking. Eventually, when the axial force dropped below 2 N,

indicating that matrix relaxation was nearly complete, G′ stabilized and reached a plateau (green region).

Similar trends have been reported for covalently crosslinked hydrogels prepared from other biopolymers [3–5]. Based on these findings, gelation time for ε-PL/HA hydrogels was defined as the initial point at which the G′ curve became flat and remained constant. The corresponding gelation times were determined as 34, 41, and 46 minutes for the 50:50, 60:40, and 70:30 wt% formulations, respectively. The observed differences are attributed to variations in the molar mass of ε-PL. Although crosslinking density was comparable across all hydrogel compositions, uncrosslinked ε-PL chains may have contributed to physical entanglement within the network, delaying both chemical crosslinking and the overall gelation process [6].

[1] Rubina, A. Injectable Mineralized Sr-Hydroxyapatite Nanoparticles-Loaded ε-Polylysine-Hyaluronic Acid Composite Hydrogels for Bone Regeneration. *Int. J. Biol. Macromol.,* **2024**, *280*, 135703. https://doi.org/10.1016/J.IJBIOMAC.2024.135703.

[2] Rebenda, D. On the Dependence of Rheology of Hyaluronic Acid Solutions and Frictional Behavior of Articular Cartilage. *Mater., 2020, Vol. 13, Page 2659* **2020**, *13* (11), 2659. https://doi.org/10.3390/MA13112659.

[3] Moubarik, A. Cornstarch and Tannin in Phenol–Formaldehyde Resins for Plywood Production. *Ind. Crops Prod.,* **2009**, *30* (2), 188–193. https://doi.org/10.1016/J.INDCROP.2009.03.005.

[4] Kowalczuk, K. Self-Degrading Multifunctional PEG-Based Hydrogels—Tailormade Substrates for Cell Culture. *Macromol. Biosci.,* **2024**, *24* (5), 2300383. https://doi.org/10.1002/MABI.202300383.

[5] Perez-robles, S. HPMC Hydrogel Formation Mechanisms Unveiled by the Evaluation of the Activation Energy. *Polym.,* **2022**, *14* (3), 635. https://doi.org/10.3390/POLYM14030635.

[6] Ramli, H. Basic Principle and Good Practices of Rheology for Polymers for Teachers and Beginners. *Chem. Teach. Int.,* **2022**, *4* (4), 307–326. https://doi.org/10.1515/CTI-2022-0010.

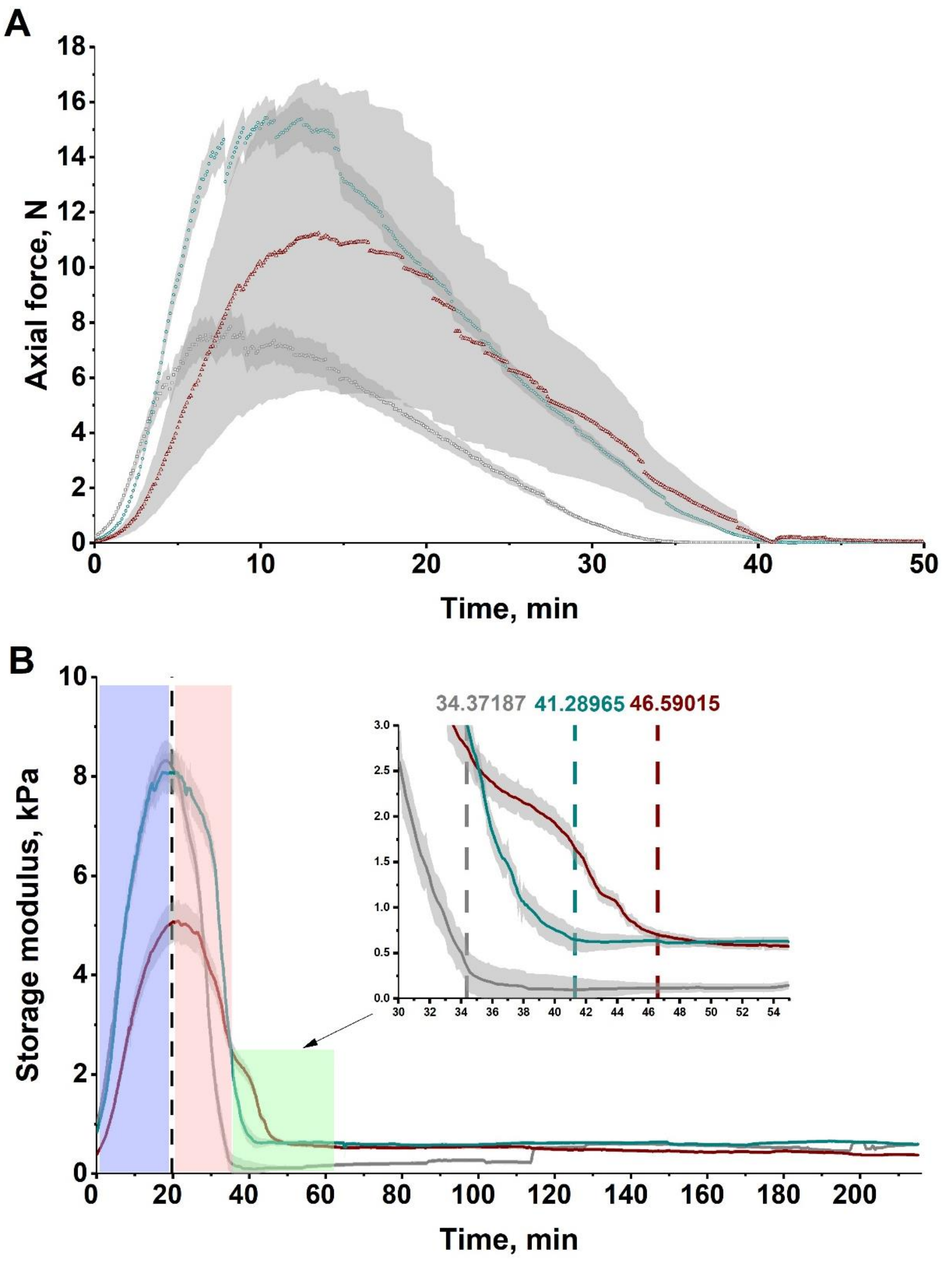

Figure S1. *Representative curves of time sweep studies.* ***(A)*** *Axial force as a function of time;* ***(B)*** *Storage modulus as a function of time. The blue region corresponds to the chemical crosslinking reaction, indicated by the rapid increase in storage modulus. The red region reflects hydrogel network rearrangement and reorganization, shrinkage, and decreased storage modulus. The green area represents matrix relaxation, where the storage modulus reaches a plateau, marking the completion of gelation. Gelation time was defined as the point*

where the decreasing storage modulus curve levels off and becomes constant. Time sweep measurements were performed in oscillatory mode, with hydrogels tested immediately after synthesis using a 1300 µm gap, 0.2% strain, 1 Hz frequency, and 37 °C. Three replicates were measured to ensure reproducibility.

S.1.2. Swelling behaviour and structural stability studies of ε-PL/HA hydrogels

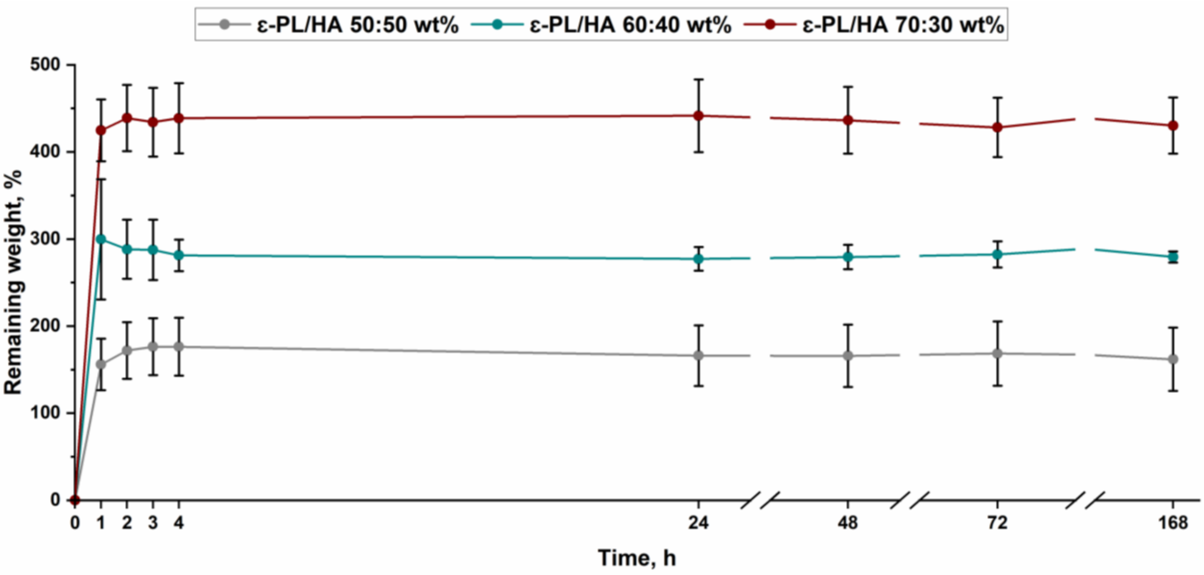

***Figure S2**. Swelling capacity and structural stability of ε-PL/HA hydrogels over 168 h. Remaining weight (%) was measured at 1, 2, 3, 4, 24, 48, 72, 96, and 168 h. Data represent mean ± SD (n=3) for each hydrogel series.*

S.1.3. Anti-biofilm activity of ε-PL/HA hydrogels via Live/Dead and viability assay

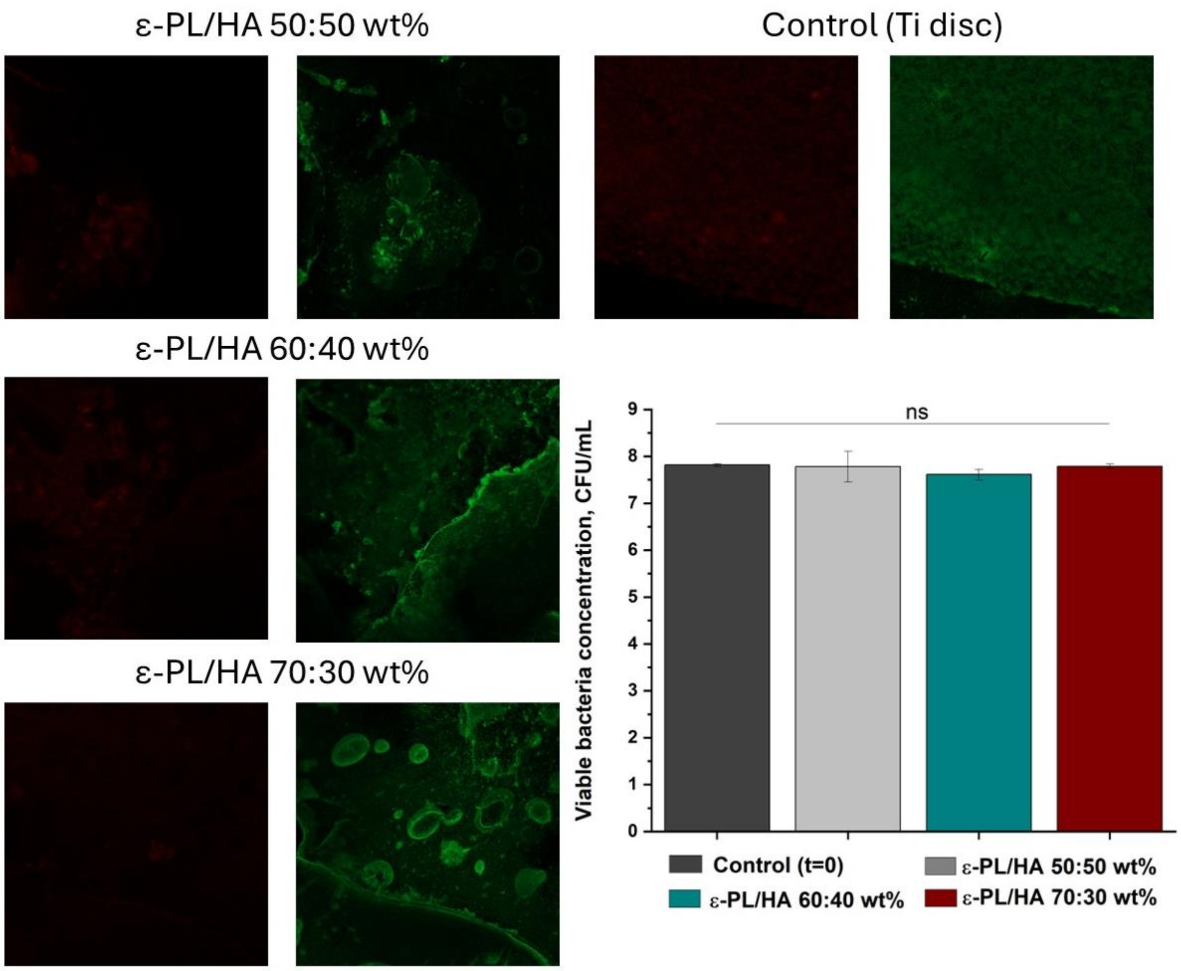

Figure S3*. Live/Dead and viability assay for ε-PL/HA hydrogels against S. aureus. Live/Dead microscopical images (scale 200 µm) illustrating bacteria attaching after 72 h incubation on the hydrogel surface. Red fluorescent dye represents dead bacteria, while Green dye represents viable bacteria found on the hydrogel surface. Quantitative graph represents viable bacteria concentration after 72 h, compared with the initial bacteria suspension concentration. Data are expressed as log viable bacteria in CFU (mean ± SD, n=3). Statistical analysis was performed by one-way ANOVA: ns - >0.05, * - <0.05, ** - <0.01, *** - <0.005 and **** - <0.001.*
